# See the (In)Visible: Infrared Thermography for Characterizing Thermal Patterns Associated with Peripheral Arterial Disease and Diabetes Mellitus

**DOI:** 10.3390/medsci14040487

**Published:** 2026-08-16

**Authors:** Slobodan Tanasković, Aleksandra Milačić, Nikola Ružić, Stefan Graovac, Jovan Petrović, Milovan Bojić, Bojan Božić

**Affiliations:** 1 Institute for Cardiovascular Diseases “Dedinje”, 11040 Belgrade, Serbia; 2 Faculty of Medicine, University of Belgrade, 11000 Belgrade, Serbia; 3 School of Electrical Engineering, University of Belgrade, 11000 Belgrade, Serbia; 4 Faculty of Physics, University of Belgrade, 11000 Belgrade, Serbia; 5 Russian Academy of Sciences, Moscow 119991, Russia; 6 Institute of Physiology and Biochemistry “Ivan Djaja”, Faculty of Biology, University of Belgrade, 11000 Belgrade, Serbia

**Keywords:** thermography, peripheral arterial disease, diabetes mellitus

## Abstract

**Background/Objectives**: Peripheral arterial disease (PAD) and diabetes mellitus (DM) may produce different and overlapping plantar thermal patterns. This study descriptively characterized whole-foot temperature-distribution patterns in clinically defined healthy, PAD, DM, and combined PAD_DM cohorts. **Methods**: This single-center, exploratory, cross-sectional observational study included 73 participants: healthy controls (*n* = 21), isolated PAD (*n* = 13), isolated DM (*n* = 19), and combined PAD_DM (*n* = 20). Right- and left-foot temperature distributions were processed separately for each participant and interpreted as one paired bilateral thermal footprint using patient-level histograms, representative bilateral kernel density estimation (KDE) profiles, and a cohort-derived deterministic rule-based model. The model was developed from the present cohort and was not evaluated as an independent diagnostic classifier. Results: Among healthy controls, 11 of 21 participants (52.4%) were assigned to healthy and 8 of 21 (38.1%) to borderline healthy categories. In the DM cohort, 7 of 19 participants (36.8%) were assigned to DM, 3 of 19 (15.8%) to borderline healthy/suspected DM or controlled DM, 3 of 19 (15.8%) to DM suspected PAD, and 5 of 19 (26.3%) to PAD_DM. In the combined PAD_DM cohort, 15 of 20 participants (75.0%) were assigned to PAD_DM. The isolated PAD cohort was the most heterogeneous: 6 of 13 participants (46.2%) were assigned to PAD_DM, while none were assigned to the isolated PAD output. **Conclusions**: This exploratory, cross-sectional study demonstrated that whole-foot plantar temperature distributions can descriptively characterize differences among healthy individuals, patients with isolated DM, and those with combined PAD and DM. While healthy controls generally demonstrated symmetric thermal footprints, the DM cohort frequently exhibited globally elevated and compact temperature-distribution profiles, while mixed asymmetric patterns were common in participants with combined PAD and DM. In contrast, the isolated PAD cohort was characterized by pronounced thermal heterogeneity.

## 1. Introduction

Peripheral arterial disease (PAD) is a prevalent circulatory disorder characterized by reduced blood flow to the lower extremities, leading to intermittent claudication, numbness, weakness, and coldness in the affected limb. Globally, PAD affects over 230 million individuals [1]. Early detection is essential to prevent severe complications, including ulcers, infections, and gangrene, which may necessitate amputation. Furthermore, timely intervention can alleviate symptoms, enhance mobility, and improve overall quality of life [2]. Moreover, PAD is a strong marker of systemic atherosclerotic disease and is associated with an increased risk of cardiovascular events, including myocardial infarction and stroke, underscoring the importance of early identification and treatment [3].

Diabetes mellitus (DM) and PAD are major, distinct contributors to lower-extremity vascular impairment. While PAD represents a macrovascular obstructive disease, DM is associated with complex microvascular, autonomic, and metabolic alterations. These two conditions frequently coexist, compounding the impairment of peripheral perfusion and increasing the risk of severe complications like tissue loss and infection [4,5]. Characterizing the effects of PAD and DM, both individually and in combination, on the baseline thermal characteristics of the foot may therefore provide valuable insights into peripheral tissue status and support the development of objective, non-invasive assessment methods.

Infrared (IR) thermography has emerged as a promising non-invasive imaging modality for evaluating peripheral tissue status by providing spatial maps of skin temperature that may indirectly reflect underlying perfusion and metabolic processes [6,7]. While thermal imaging has been explored for detecting localized inflammation or regional abnormalities, many existing applications rely on single-point or regional region-of-interest (ROI) measurements [8,9,10,11].

The integration of thermal imaging into clinical practice holds promise for enhancing wound assessment and improving patient outcomes [12]. In parallel, the application of machine learning (ML) to thermographic data has been explored as a means of improving the assessment of diabetic foot complications, highlighting the expanding role of digital imaging and computational methods in peripheral vascular and metabolic disease assessment [13].

Despite these advances, many existing clinical applications of thermography rely on single-point temperature measurements at predefined anatomical landmarks or summary statistics derived from broad ROIs [6,14]. Although such approaches may be useful for assessing localized abnormalities, they substantially reduce the spatial information contained within a thermal image and may therefore fail to capture the complex and heterogeneous temperature patterns associated with vascular, metabolic, autonomic, and microcirculatory disturbances.

The key methodological novelty of the present study is the consideration of the entire visible plantar temperature field as a continuous distributional object rather than as a set of isolated temperature measurements. By analyzing pixel-level temperature data from each foot using patient-level histograms and kernel density estimation (KDE), this approach preserves the full spectrum of thermal variation and heterogeneity within the plantar surface. Such distribution-based characterization may provide a more comprehensive representation of the thermal footprint associated with different peripheral vascular and metabolic conditions.

The physiological mechanisms regulating foot skin temperature are complex and highly variable. Resting thermography in PAD has notable limitations, as low resting metabolic demands and compensatory collateral circulation can maintain baseline skin temperature, masking significant arterial stenoses [15,16]. To unmask these latent deficits, dynamic provocative testing, such as local cooling or standardized exercise, is often used to demonstrate delayed rewarming or a post-exercise drop in distal temperature due to proximal blood diversion [17,18,19].

In diabetic patients, skin temperature is shaped by competing pathophysiological pathways. Autonomic neuropathy leads to sympathetic denervation and arteriovenous shunting, producing a paradoxically warm foot [6,20]. Conversely, macrovascular ischemia cools the tissue, while microvascular dysfunction impairs local vasoactive responses [14,15]. At a localized level, side-to-side plantar temperature asymmetries exceeding 2.2 °C, driven by neuropathic microtrauma and subclinical inflammation, serve as validated early predictors of diabetic foot ulcer (DFU) risk [7,12]. When macrovascular ischemia, microvascular dysfunction, and neuropathy coexist, they create highly heterogeneous and overlapping thermal patterns. Capturing this complexity requires moving beyond single-point or regional average measurements to evaluate the entire plantar surface as a continuous temperature distribution

Previous studies on infrared thermography have commonly focused on diabetic foot ulceration, wound monitoring, infection, or localized temperature differences. Resting thermography in PAD is limited by disease severity, anatomical distribution, laterality, collateral circulation, distal runoff, and microcirculatory adaptation, while DM may influence temperature through several metabolic, inflammatory, autonomic, and microvascular mechanisms [6,14,15,19].

Based on the differing macrovascular, microvascular, and neuropathic mechanisms of PAD and DM, we formulated three prespecified hypotheses regarding their whole-foot thermal footprints before data analysis: 1. Isolated PAD would be associated with lower mean plantar temperatures and more pronounced bilateral (right–left) asymmetry, reflecting localized macrovascular arterial obstruction. 2. Isolated DM would be associated with higher mean plantar temperatures and narrower (more compact) temperature-distribution widths, reflecting autonomic neuropathy-driven hyperperfusion and a reduced intra-foot thermal span. 3. Combined PAD and DM would demonstrate a transitional, mixed thermal phenotype with overlapping features, combining elevated absolute temperatures with prominent spatial asymmetries.

The primary objective of this exploratory study was to compare whole-foot plantar temperature distributions across four clinically defined groups: healthy controls, patients with isolated PAD, patients with isolated DM, and patients with combined PAD and DM. Specifically, we aimed to characterize the distinct effects of PAD, DM, and their coexistence on whole-foot thermal footprints and to summarize these patterns using a transparent, cohort-derived descriptive model. This exploratory analysis was not intended to diagnose diabetic neuropathy, predict clinical outcomes, or replace established gold-standard vascular, metabolic, or neurological assessments. Rather, it was designed to investigate whether whole-foot temperature distributions may provide a more comprehensive characterization of peripheral thermal phenotypes associated with PAD and DM.

## 2. Materials and Methods

### 2.1. Study Design and Participant Groups

This single-center, exploratory, cross-sectional observational study was designed to characterize descriptive lower-extremity thermal-distribution patterns in participants with clinically defined PAD, DM, and their combined presentation (PAD_DM). Participants were prospectively enrolled upon clinical admission to the Clinic for Vascular Surgery at the Institute for Cardiovascular Diseases “Dedinje” during a study period spanning from March 2026 to May 2026. Healthy participants were prospectively recruited from volunteers during the same period to serve as a descriptive reference group. The final cohort comprised 73 participants: healthy controls (*n* = 21), patients with PAD (*n* = 13), patients with DM (*n* = 19), and patients with combined PAD and DM (*n* = 20) (Table 1).

All participants with PAD and/or DM were recruited among patients admitted to the Clinic for Vascular Surgery for elective vascular treatment, including open surgical or endovascular procedures. Detailed clinical documentation was available for all patients, including vascular imaging studies and angiographic findings, enabling correlation of thermal imaging parameters with the known vascular status of the lower extremities.

The diagnosis of PAD was established by an experienced vascular surgeon based on medical history, clinical examination, and multidetector computed tomography (MDCT) angiography findings. The diagnosis of DM had been previously established by an endocrinologist before hospital admission, and all DM patients had documented endocrinological follow-up and ongoing antidiabetic therapy.

The healthy control group consisted of young adult volunteers, primarily physicians, nurses, and non-medical hospital personnel, without a history of DM, PAD, or other significant vascular disorders. All healthy participants had normal laboratory findings and available medical documentation confirming the absence of the studied conditions.

Because this was designed as a pilot-scale, exploratory study, no formal, a priori statistical power calculation was performed to determine the sample size. Instead, a pragmatic convenience target of approximately 20 participants per group was planned to gather baseline descriptive data. The final group distribution reflected the real-world clinical characteristics of our vascular surgery population. The smaller number of patients in the isolated PAD-only group (*n* = 13) reflects the strong association between DM and PAD, as many patients presenting with PAD also had a documented history of DM and were therefore assigned to the combined PAD_DM group (*n* = 20).

Participants included in the DM-only group (*n* = 19) were patients with a documented history of DM who were admitted to the Clinic for Vascular Surgery primarily for carotid artery surgery, where the presence of lower-extremity PAD was excluded by clinical examination and Doppler ultrasonography. The Doppler ultrasound exclusion criteria required the presence of normal triphasic or biphasic velocity waveforms in the common femoral, superficial femoral, popliteal, and pedal (anterior and posterior tibial) arteries, with no evidence of hemodynamic stenosis (defined as a peak systolic velocity ratio ≥2.0 or a focal lumen reduction > 50%). Ankle-brachial index (ABI) was also measured to confirm a resting index between 0.90 and 1.30. While these patients represented a subset with established atherosclerosis due to their carotid stenosis, they remained free of lower-extremity PAD as verified by clinical examination, normal ABIs, and Duplex ultrasound (DUS). Consequently, this cohort remained eligible for evaluating diabetes-specific thermal footprints without the confounding influence of active lower-limb ischemia. Following confirmation of eligibility, these patients were invited to participate in the study.

Exclusion criteria for all participant groups included the presence of active, spreading local lower-extremity infections (e.g., wet gangrene, active cellulitis, or deep abscesses), acute osteomyelitis, systemic infection, active fever (body temperature > 37.5 °C), severe bilateral lower-extremity edema, or the ongoing use of acute intravenous antibiotic therapy at the time of imaging. Any chronic, stable, non-infected trophic lesions (such as localized dry digital gangrene or stable chronic ischemic ulcers) were documented.

All participants provided written informed consent before enrolment. The study population was therefore intentionally structured to include comparative cohorts of patients with isolated DM, isolated PAD, their combined presentation, and healthy controls, enabling comparison of thermal footprint characteristics across different clinical conditions.

Thermal imaging was performed to characterize recurring and transitional foot thermal-distribution patterns associated with clinically defined PAD, DM, and their combined presentation. By comparing the thermal profiles of the clinical cohorts with the range of variation observed in the healthy reference group, the study evaluated relationships between whole-foot thermal descriptors and the participants’ established clinical status. The analysis was descriptive and was not designed to establish diagnostic performance.

For each participant, the thermal profiles of the right and left lower extremities were analyzed separately and subsequently evaluated as a paired bilateral thermal footprint. This paired structure enabled the assessment of both absolute temperature characteristics and right–left thermal asymmetry.

### 2.2. Thermal Imaging Protocol

All thermal imaging was performed using a high-resolution InfiRay S600 thermal camera (Yantai MicroSensors Co., Ltd., Yantai, China). The camera features an uncooled Vanadium Oxide (VOx) microbolometer detector, a spectral range of 8–14 μm, a thermal sensitivity (Noise Equivalent Temperature Difference, NETD) of <40 mK (<0.04 °C) at 30 °C, an infrared spatial resolution of 640 × 512 pixels, and a manufacturer-specified measurement accuracy of ±2 °C or ±2% of the reading. During image acquisition, the camera emissivity setting was set to 0.98 (the standard emissivity of human skin), and the reflected apparent temperature was compensated based on the ambient room temperature. To ensure reproducibility, a standardized clinical and environmental protocol was strictly followed. Imaging was performed in a dedicated, draft-free, temperature-controlled room maintained at an ambient temperature of 20 ± 1 °C and a relative humidity of 40–60%. To control for diurnal temperature fluctuations, all acquisitions were performed during the morning hours (between 9:00 AM and 12:00 PM).

Before imaging, participants were instructed to refrain from smoking, performing strenuous physical exercise, and consuming caffeinated beverages or hot meals for at least 2 h. Compliance was verbally verified before imaging. All imaging was performed before any scheduled vascular interventions.

Upon entering the examination room via slow walking or wheelchair (to minimize lower-extremity thermal generation from exertion), participants immediately removed their shoes, socks, and any bandages. They then underwent a 15 min resting acclimatization period in a supine position on an examination table, with their lower legs extended and feet supported at the ankles to prevent contact of the plantar surface with the bedding.

Following acclimatization, thermal images of the plantar surfaces of both feet were captured. The camera was positioned on a tripod at a distance of approximately 1.5 m from the soles of the feet, oriented perpendicular to the plantar plane. The camera’s automatic internal shutter calibration was executed immediately before each image capture to ensure thermal stability and accuracy.

### 2.3. Thermal Data Extraction and Preparation

For each participant, thermal data from the right and left feet were exported separately into Comma-Separated Values (CSV) files. Each CSV contained temperature values from the plantar-foot ROI available in the source thermography dataset. The analytical target was the visible plantar surface rather than the surrounding background or lower leg, and the same whole-foot extraction principle was applied to the right and left sides. The computational workflow converted each temperature matrix into a one-dimensional numerical vector and removed missing values before further processing. Thus, each foot was represented as an empirical temperature distribution rather than as a single-point measurement, and the participant remained the primary unit of analysis.

### 2.4. Pre-Processing of Temperature Distributions

Before feature extraction, a fixed percentile-based trimming step was applied uniformly to every foot distribution: the lower and upper 0.5% of temperature values were removed. This preprocessing choice was established before derivation of the thermal-pattern boundaries and was not adjusted by participant or clinical group. It was intended to reduce the influence of isolated sensor artifacts, reflections, edge pixels, and other extreme values while retaining the broad whole-foot thermal span.

For a given temperature distribution:

*T* = {*T*_1_, *T*_2_, …, *T_n_*}

The trimmed distribution was defined as:

*T*_*trim* = {*T_i_*|*P*_0.5_(*T*) ≤ *T_i_* ≤ *P*_99.5_(*T*)}

where *P*_0.5_(*T*) and *P*_99.5_(*T*) represent the 0.5th and 99.5th percentiles of the temperature distribution, respectively. This trimming procedure was used to limit the effect of isolated thermal artifacts, sensor noise, reflections, and potential pixels outside the relevant anatomical region.

### 2.5. Feature Extraction

The absolute median and mean temperatures of the right and left feet were retained for descriptive inspection but were not included as primary decision variables in the rule hierarchy. This choice was made because absolute skin temperature is highly sensitive to systemic, non-pathological confounders such as age, sex, BMI, and ambient room conditions. To mitigate these confounding effects, we prioritized relative, intra-individual features. For each trimmed temperature distribution, the following parameters were calculated:

(1) Mean temperature

*μ* = (1/*N*) *Σ_i_*_=__1_*N*
*T_i_*

where *N* is the number of temperature values after trimming;

(2) Distribution width

*W* = *T_max* − *T_min*

where *T_max* and *T_min* are the maximum and minimum values of the trimmed temperature distribution.

(3) Median temperature

$$\tilde{T}=median(T)$$

The median temperature was calculated as an auxiliary descriptive parameter. However, it was not used as a decision variable in the final rule-based classifier.

Thus, the primary features used for classification were:

*μ_R*, *μ_L*, *W_R*, *W_L*

where *R* and *L* denote the right and left lower extremities, respectively.

Bilateral thermal asymmetry was quantified as the absolute difference between the mean temperatures of the right and left extremities:

Δ*μ* = |*μ_R* − *μ_L*|

Accordingly, each participant was represented by a bilateral thermal-feature vector:

*x* = [*μ_R*, *μ_L*, *W_R*, *W_L*, Δ*μ*]

The median values of the right and left feet were retained for descriptive inspection but were not included in the rule hierarchy. The trimmed max-min width was selected because it provides an immediately interpretable measure of the total thermal span within one plantar field and can reflect the coexistence of warmer and cooler regions in the same foot. Bilateral asymmetry based on whole-foot mean temperature was intentionally chosen as a simple paired descriptor. These deliberately transparent features do not exhaust the spatial information in the thermogram and may miss abnormalities confined to specific anatomical subregions.

### 2.6. Bilateral Thermal-Footprint Representation

The right and left lower-extremity temperature distributions were aligned by the participant. After individual feature extraction, each participant was represented using a paired right–left thermal profile (Table 2). This paired representation allowed the analysis to account for both the absolute thermal level of each extremity and the degree of thermal asymmetry between the two sides.

### 2.7. Exploratory Distribution Analysis

KDE was used as an exploratory patient-level visualization. A Gaussian KDE was calculated separately for the trimmed right-foot and left-foot temperature vectors of each participant using the same implementation-default Scott-type bandwidth rule. Raw pixels from different participants were not concatenated, and individual pixels were not treated as independent observations across the cohort.

**Figure 1:**
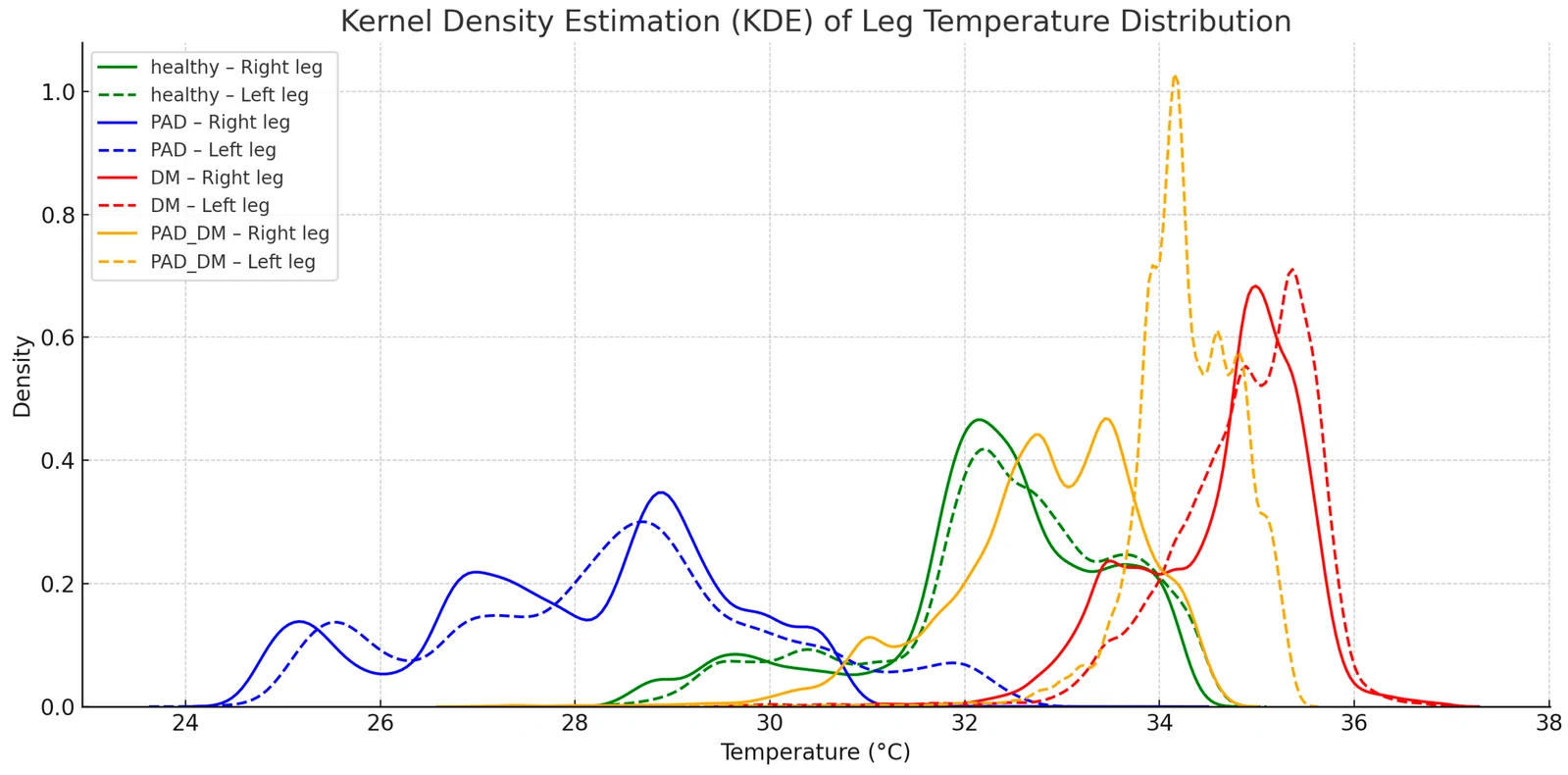
Representative patient-level KDE profiles of right- and left-foot temperature distributions. Each pair of curves corresponds to one representative participant from the healthy, PAD, DM, or PAD_DM group. Right and left feet were processed separately and retained as a paired bilateral profile; data from different participants were not pooled.

Figure 1 presents the paired right- and left-foot KDE profiles of one representative participant from each of the four basic clinical groups. The curves are illustrative examples of individual bilateral thermal footprints, not group averages. They visualize curve position, distribution width, and right–left separation within a participant and support interpretation of the patient-level descriptors used by the rule-based model.

In addition to KDE visualization, two-dimensional feature-space plots were generated to inspect the relationship between mean temperature, temperature-distribution width, and bilateral asymmetry.

The following feature relationships were considered:

*W* *vs.* *μ* Δ*μ vs.* (*W* − *W_ref*)

where the reference distribution width based on our investigated cohort was set to:

*W_ref* = 5 °C

These plots were used for methodological assessment of the extracted features and for visual inspection of the right–left thermal-profile structure.

### 2.8. Rule-Based Thermal Classification

A deterministic rule-based model was implemented to assign each participant to an interpretable thermal-pattern category. The model used paired bilateral features extracted from the right and left lower extremities and was intended to summarize the recurring and transitional patterns observed in this cohort.

The decision variables used by the classifier were:

*μ_R*, *μ_L*, *W_R*, *W_L*, Δ*μ*

where *μ_R* and *μ_L* are the mean temperatures of the right and left lower extremities, *W_R* and *W_L* are the corresponding temperature-distribution widths, and Δ*μ* is the absolute right–left mean-temperature difference.

The rule-based model was developed during exploratory analysis of the present cohort. The temperature, bilateral-asymmetry, and distribution-width boundaries were not imported from established clinical guidelines and were not derived using receiver-operating-characteristic optimization or another procedure intended to maximize apparent classification performance. Instead, they were selected by examining recurring transitions in the patient-level distributions of mean temperature, temperature-distribution width, and bilateral mean-temperature difference across the four clinically defined cohorts.

Following preprocessing and feature extraction, patient-level histograms, individual bilateral KDE profiles, and two-dimensional feature-space plots were inspected to identify broad low-, intermediate-, and high-temperature regions and to characterize transitions between relatively symmetric, asymmetric, broad, and compact thermal distributions. The temperature values of 27 °C, 32 °C, and 33 °C were consequently used as descriptive boundaries between the principal temperature regions observed in this cohort. Similarly, bilateral-difference values of 0.25 °C, 0.40 °C, and 0.50 °C were used to distinguish very small, intermediate, and more pronounced right–left differences within the observed distributions.

A reference distribution width of W_ref = 5.0 °C was used to distinguish relatively broad thermal distributions from more compact distributions. In the low-temperature asymmetric branch, this requirement was relaxed by the cohort-derived parameter pad_band = 0.8 °C, resulting in a relaxed width boundary of W_ref − pad_band = 4.2 °C. This relaxation was introduced to prevent low-temperature asymmetric patterns with only mildly reduced thermal width from being automatically assigned to a mixed PAD_DM branch.

The healthy cohort was used as a descriptive reference for the range of thermal variation observed in participants without clinically diagnosed PAD or DM, rather than as an assumption that healthy thermal distributions must be uniform. Accordingly, the framework retained borderline and transitional outputs instead of forcing every participant into a single disease-equivalent category.

All boundaries should be interpreted as cohort-derived descriptive band limits rather than clinical diagnostic cut-offs. Their purpose was to summarize the thermal-pattern continuum observed in this exploratory dataset using simple and traceable rules. Because the same cohort informed and received these rules, the outputs are descriptive by construction and cannot be used to estimate out-of-sample sensitivity, specificity, predictive value, or accuracy.

The model used the following cohort-derived reference parameters:

*W_ref* = 5.0 °C *pad_band* = 0.8 °C

The ordered decision hierarchy first identifies healthy and borderline healthy patterns, then evaluates the intermediate-temperature region according to bilateral asymmetry and width, followed by the DM, DM suspected PAD, PAD_DM, PAD and suspected DM, and PAD branches. The first satisfied rule determines the participant-level output. The complete equations, thresholds, and branch-specific codes are retained in the Supplementary Information (Supplementary S1–S4, Supplementary Table S2). The category names are intentionally concise and clinically intuitive; they denote thermal-pattern interpretations and do not independently establish a diagnosis, glycemic control, or disease severity.

No internal cross-validation was performed because the rule system was not used to estimate predictive performance. A random train-validation split in this small exploratory cohort would reduce the descriptive basis of each subgroup without converting empirically observed boundaries into independently validated clinical cut-offs. Prospective recalibration and external validation are therefore required before performance metrics can be meaningfully reported.

The classification rules were applied using vectorized logical conditions. Each participant was assigned a numerical code and a descriptive thermal-pattern label according to combinations of absolute mean temperature, bilateral thermal asymmetry, and temperature-distribution width. These assignments are reported as descriptive outputs and not as independently validated diagnoses.

The rule-based approach was selected because it provides direct interpretability: each assigned pattern can be traced back to explicit thermal criteria involving temperature level, distribution width, and right–left asymmetry. An overview of the framework and the complete hierarchy of cohort-derived rules is provided in the Supplementary Information (Supplementary S4). The reported outputs should be interpreted as a transparent summary of the observed thermal-pattern continuum rather than as measures of diagnostic accuracy.

### 2.9. Descriptive Reporting and Unit of Analysis

The participant was the unit of analysis. Each participant contributed one paired bilateral thermal profile composed of separately processed right- and left-foot distributions. Pixel values were used to characterize within-foot distributions but were not treated as independent cohort observations, and the two feet were not pooled into a single group-level temperature distribution.

Because the objective was descriptive, patient-oriented thermal phenotyping rather than hypothesis testing of group separability, the primary results are reported as the number and percentage of participants assigned to each original rule-based output category. Per-foot descriptive summaries for *μ_R*, *μ_L*, median temperature, *W_R*, *W_L*, and Δ*μ* are provided in the Supplementary Information solely to contextualize the observed ranges without replacing the paired patient-level interpretation (Supplementary S5, Supplementary Tables S1 and S3).

No diagnostic-accuracy metrics, multivariable group model, or internal cross-validation estimate was calculated. Such analyses would imply a predictive validation objective that the present cohort-derived framework was not designed to address. All 73 participants had complete values for the principal thermal descriptors; no thermal-feature imputation was performed.

## 3. Results

### 3.1. Clinical Characteristics of Study Cohorts

#### 3.1.1. Healthy Controls

The healthy control group was described previously and demonstrated normal clinical, laboratory, and vascular parameters without evidence of DM or PAD.

#### 3.1.2. PAD Group

The PAD cohort included 13 patients with symptomatic PAD without DM. Clinical presentation ranged from intermittent claudication to rest pain and chronic limb-threatening ischemia. MDCT angiography demonstrated advanced multilevel atherosclerotic disease, predominantly affecting the aortoiliac and femoropopliteal segments, with frequent popliteal and infrapopliteal involvement. Multivessel tibial artery disease was common, and previous endovascular interventions were documented in a subset of patients. Overall, this cohort represented patients with isolated PAD and varying degrees of lower-limb ischemia.

#### 3.1.3. DM Group

The DM cohort consisted of 19 patients with established DM and no evidence of PAD on clinical examination and Doppler ultrasonography findings. Most patients, 18 of 19 (94.7%), were treated with oral antidiabetic therapy; 1 of 19 (5.3%) received semaglutide, while none of the 19 patients was receiving insulin treatment. These patients represented a diabetic population without clinically significant lower-extremity arterial disease and served as a reference group for evaluating the isolated effect of diabetes on thermal footprint characteristics.

#### 3.1.4. PAD_DM Group

The PAD_DM cohort consisted of 20 patients with combined DM and PAD, representing the most clinically complex vascular group. Intermittent claudication was the most common presentation, although a substantial proportion presented with rest pain or chronic limb-threatening ischemia, including tissue loss, ulceration, gangrene, and DFUs. MDCT angiography revealed extensive multilevel arterial disease involving the aortoiliac, femoropopliteal, and infrapopliteal segments, with impaired distal runoff frequently observed. Previous vascular interventions, including bypass grafting and endovascular stenting, were also common, as in the PAD-only group. This cohort represented the combined effects of diabetes and advanced PAD on lower-extremity perfusion and tissue status (Table 3).

### 3.2. Infrared Thermography Findings

Infrared thermography demonstrated descriptive differences in foot thermal-distribution patterns across the four study groups. The analysis integrated representative infrared images, patient-level temperature histograms, and KDE curves derived from extracted foot temperature distributions.

**Figure 2:**
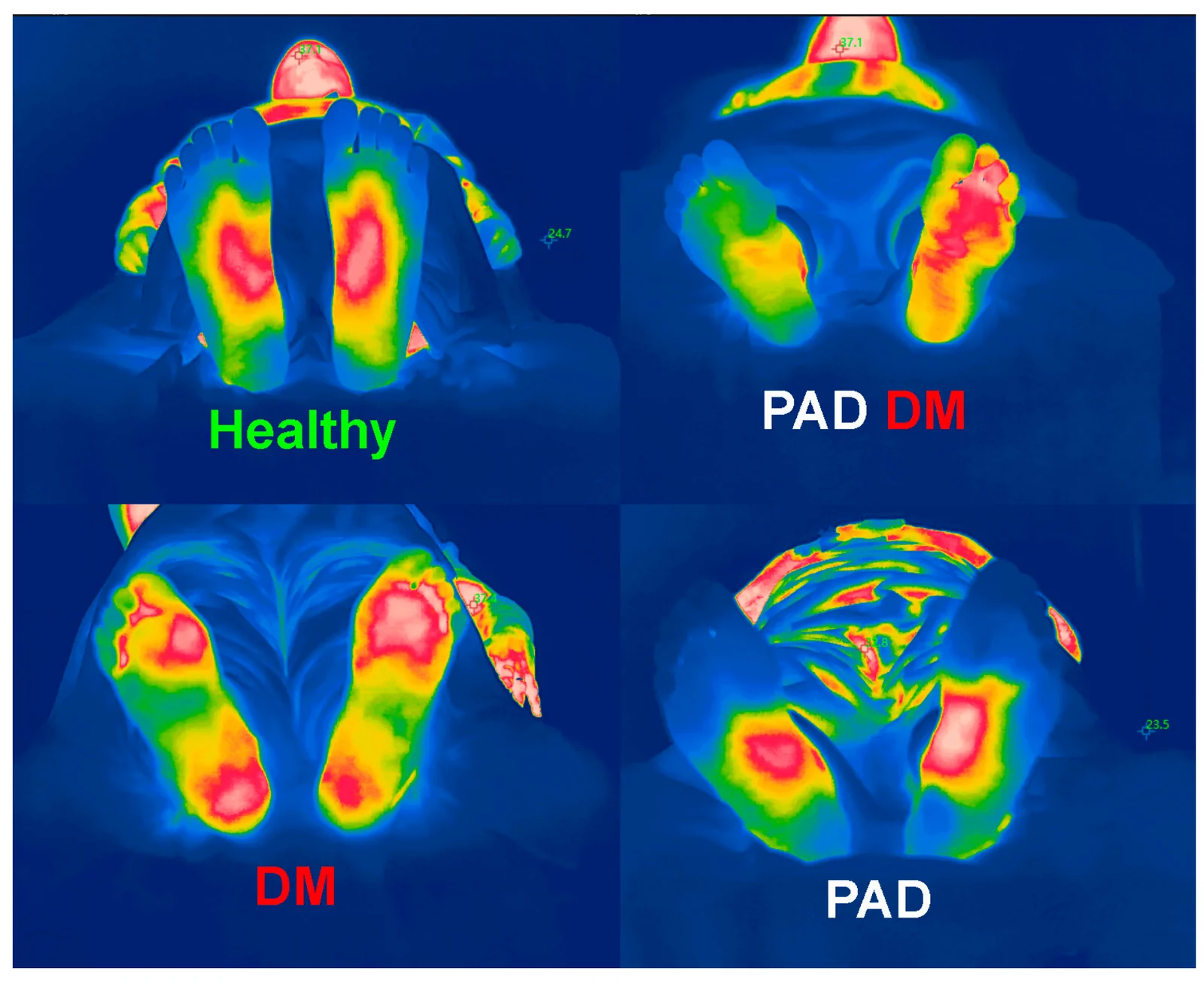
Representative infrared thermographic images of the feet. The panels are qualitative examples selected to illustrate thermal-pattern diversity; they are not matched cases and should not be used for direct quantitative comparison of color fields, positioning, or field of view. All quantitative analyses were based on exported ROI temperature values.

Given the marked heterogeneity and overlap of the thermal patterns, additional interpretative subcategories were retained to describe borderline and transitional outputs. These categories were used to summarize intermediate thermal presentations and were not treated as replacement clinical diagnoses or as evidence of classification accuracy. Figure 2 shows representative infrared thermograms from each group, illustrating qualitative differences in foot thermal footprints across the clinically defined cohorts.

The visual thermal patterns differed between clinical categories, indicating that the spatial distribution of foot temperature contains clinically relevant information beyond a single-point temperature measurement. Healthy controls generally presented a balanced bilateral thermal pattern. In contrast, PAD cases showed thermographic features compatible with reduced distal perfusion, while DM cases demonstrated a more globally elevated and comparatively compact thermal appearance. The combined PAD_DM group exhibited a mixed phenotype, reflecting the coexistence of vascular insufficiency and diabetes-related thermal alteration.

For each participant, the temperature values extracted from the right and left feet were used to construct patient-level histograms. These histograms provided the empirical basis for subsequent distributional analysis and allowed the thermal footprint to be evaluated in terms of central tendency, distribution width, and bilateral symmetry. KDE curves were generated separately for each foot and used to obtain a smoothed representation of that participant’s paired right- and left-foot temperature distributions.

The KDE curves illustrate selected patient-specific bilateral thermal footprints and are not intended as group averages or inferential summaries. Their purpose is to show how curve position, width, and right–left separation contributes to the participant-oriented interpretation.

#### 3.2.1. Healthy Controls

In the healthy group (*n* = 21), patient-level temperature distributions generally showed preserved bilateral symmetry together with appreciable physiological variation in absolute temperature and thermal spread. The representative bilateral KDE profile in Figure 1 illustrates closely aligned right- and left-foot curves for one healthy participant; it is not a pooled group curve.

When the cohort-derived rule-based model was applied to the clinically defined healthy group, 11 of 21 participants (52.4%) were assigned to healthy and 8 of 21 (38.1%) to a borderline healthy category. Within the latter, 4 of 21 participants (19.0%) were assigned to borderline healthy/suspected PAD and 4 of 21 (19.0%) to borderline healthy/suspected DM or controlled DM. One participant (1/21, 4.8%) was assigned to PAD and one participant (1/21, 4.8%) to DM. These findings show that the healthy reference cohort was not thermally uniform, which was one of the intended observations of including this group.

#### 3.2.2. PAD Group

The PAD group represented the smallest clinical subgroup in the cohort (*n* = 13). At the group level, PAD cases showed pronounced thermal heterogeneity, with thermal profiles ranging from relatively preserved bilateral patterns to asymmetric, DM-like, and mixed PAD_DM thermal presentations. This variability is consistent with the clinical complexity of isolated PAD assessment, where perfusion-related thermal changes may differ depending on disease severity, anatomical distribution of arterial lesions, limb involvement, collateral circulation, and local microcirculatory status.

Application of the cohort-derived rule-based model further demonstrated the heterogeneous thermal behavior of the clinically defined PAD group. Six of 13 participants (46.2%) were assigned to PAD_DM, 2 of 13 (15.4%) to PAD and suspected DM, and 1 of 13 (7.7%) to DM suspected PAD. Two of 13 participants (15.4%) were assigned to borderline healthy/suspected DM or controlled DM, 1 of 13 (7.7%) to DM, and 1 of 13 (7.7%) to healthy. None of the 13 participants was assigned to the isolated PAD output.

Therefore, within the PAD-only cohort, thermography did not reveal a uniform isolated-PAD phenotype, and the ability of the present rule-based model to identify isolated PAD as one consistent output was not demonstrated. At the same time, the observed spread across healthy, DM-like, asymmetric, and mixed categories is itself compatible with the diverse origin, anatomical distribution, laterality, collateral compensation, distal runoff, severity, previous intervention, and resting microcirculatory expression of PAD. The current dataset cannot determine how much of this variability reflects disease biology and how much reflects the selected descriptors and cohort-derived rule structure.

#### 3.2.3. DM Group

In the DM group (*n* = 19), many patient-level thermal profiles showed a shift toward higher foot temperatures, often accompanied by a comparatively reduced temperature-distribution width. The representative DM participant in Figure 2 shows a compact and elevated pair of temperature-density curves. This example is consistent with the recurring pattern observed in the DM cohort but is not presented as a pooled or average DM distribution. Potential contributions from microcirculatory, inflammatory, autonomic, or treatment-related factors remain interpretative hypotheses because they were not directly measured.

When the cohort-derived rule-based model was applied to the clinically defined DM group, 7 of 19 participants (36.8%) were assigned to DM, 3 of 19 (15.8%) to borderline healthy/suspected DM or controlled DM, and 3 of 19 (15.8%) to DM suspected PAD. Five of 19 participants (26.3%) were assigned to PAD_DM, while 1 of 19 (5.3%) was assigned to borderline healthy/suspected PAD. These outputs describe recurring elevated and compact presentations while retaining the observed overlap with asymmetric and mixed thermal patterns (Supplementary S6).

#### 3.2.4. PAD_DM Group

The PAD_DM group (*n* = 20) exhibited a complex but predominantly mixed thermal behavior among the analyzed clinical groups. At the group level, the temperature distributions frequently combined features associated with both vascular insufficiency and diabetes-related thermal alteration. Several cases showed elevated or compact thermal profiles compatible with DM-like thermal behavior, while others demonstrated marked bilateral asymmetry or side-specific thermal shifts consistent with PAD-like perfusion-related changes. This mixed distributional structure supports the interpretation that patients with combined PAD and DM cannot be represented by a purely ischemic or purely diabetic thermal profile.

When the cohort-derived rule-based model was applied to the clinically defined combined PAD_DM group, 15 of 20 participants (75.0%) were assigned to PAD_DM. One of 20 participants (5.0%) was assigned to DM suspected PAD, 2 of 20 (10.0%) to DM, 1 of 20 (5.0%) to PAD, and 1 of 20 (5.0%) to borderline healthy/suspected PAD. The dominant output was therefore the combined PAD_DM category, although the cohort retained several alternative patient-level presentations.

The complete distribution of the original rule-based output categories within each clinically defined cohort is summarized in Table 4 and Supplementary Table S4. Values are reported directly as *n* (%) within each cohort. No post hoc broad-spectrum aggregation is used as a measure of agreement, and no classification-accuracy statistic is calculated.

## 4. Discussion

This exploratory, cross-sectional study characterized whole-foot plantar thermal-distribution patterns across clinically defined healthy, isolated PAD, isolated DM, and combined PAD_DM cohorts. By treating each participant’s bilateral plantar thermal footprint as a continuous distributional object, this patient-oriented analysis yielded three principal findings: 1. The isolated DM group showed globally higher temperatures and relatively narrow, compact temperature distributions. 2. Mixed and transitional thermal patterns were highly common in the combined PAD_DM group, reflecting the overlapping, concurrent influences of diabetes-related metabolic alterations and macrovascular arterial insufficiency. 3. No consistent, uniform thermal phenotype was identified in the isolated PAD group, which demonstrated the greatest degree of thermal heterogeneity and spanned a broad range of individual presentations.

These findings were mapped using a transparent, cohort-derived rule-based model designed to descriptively summarize individual patient-level thermal variations rather than to serve as a validated diagnostic classifier. The healthy group provided a descriptive reference for the range of thermal variation in participants without clinically diagnosed PAD or DM. Eleven of 21 participants (52.4%) were assigned to healthy, 8 of 21 (38.1%) to the two borderline healthy categories, and 2 of 21 (9.5%) to PAD or DM outputs. These findings indicate that healthy whole-foot thermal distributions are relatively heterogeneous rather than uniform. Borderline outputs should not be interpreted as replacement diagnoses; they are clinically intuitive labels for transitional thermal patterns that may reflect physiological variation, subclinical change, or other individual factors.

The PAD-only group represented the most heterogeneous subgroup. None of the 13 clinically defined PAD cases were assigned to PAD, while the cohort spanned healthy, borderline, DM, DM suspected PAD, PAD and suspected DM, and PAD_DM outputs. The present framework therefore did not demonstrate an ability to identify isolated PAD as one uniform thermal category. This negative result should not, however, be interpreted without the clinical context: symptomatic PAD varies in origin, anatomical level, laterality, collateral circulation, distal runoff, severity, previous intervention, and resting microcirculatory expression. The current design cannot quantify how much of the observed variability derives from disease biology and how much from the selected whole-foot descriptors and cohort-derived branches.

This finding is clinically plausible. Although PAD is generally expected to produce lower distal temperatures or asymmetric thermal patterns due to impaired arterial perfusion, the thermal manifestation of PAD may vary substantially depending on disease severity, anatomical distribution of arterial lesions, unilateral versus bilateral involvement, collateral circulation, distal runoff, and local microcirculatory status. Furthermore, PAD is a dynamic disease, and resting thermal patterns may appear relatively preserved despite underlying hemodynamic impairment. Temperature abnormalities are likely to become more pronounced during or after physical activity, when increased metabolic demand requires augmented blood flow that cannot be adequately supplied by diseased arteries. Future studies should therefore investigate infrared thermography in combination with standardized exercise or functional testing to determine whether dynamic thermal responses improve the detection and characterization of PAD. The frequent occurrence of mixed or DM-like classifier outputs further suggests that PAD-related thermal asymmetry may overlap with compact, elevated, or otherwise altered thermal distributions usually associated with diabetes-related or microcirculatory changes. Therefore, isolated PAD appears to require cautious interpretation in distribution-based thermographic analysis, particularly when classifier outputs are considered as strict one-to-one equivalents of clinical diagnoses.

To contextualize these findings, it is essential to define the potential clinical value of whole-foot infrared thermography not as a replacement for, or an ‘earlier’ screen than, established diagnostic modalities, but as a rapid, non-contact physiological adjunct. Standard clinical examinations each possess distinct limitations: the ABI is frequently falsely elevated or normal in patients with diabetes due to medial arterial calcification and non-compressible vessels [14]; TBI is less affected by calcification but requires specialized equipment and cuffs; DUS is highly operator-dependent and focuses on localized anatomical flow velocities rather than global skin perfusion; and clinical neuropathy assessments (such as monofilament or tuning fork testing) evaluate somatic sensory loss but cannot objectively measure autonomic vasomotor dysfunction. Whole-foot thermography adds value by providing a rapid, objective, zero-contact spatial map of the entire plantar surface, reflecting the net physiological output of both macrovascular perfusion and autonomic-microvascular thermoregulation. However, given the high degree of thermal heterogeneity observed in our isolated PAD cohort, thermography cannot be proposed as an ‘earlier’ or more sensitive screening test than conventional examinations. Instead, its clinical utility lies in its role as an objective, first-line visual flag. In outpatient or primary care settings where full vascular or neurological assessments are not routinely available, detecting an abnormal, highly asymmetric (Δμ), or globally compact and hyperthermal (W) plantar footprint could provide immediate, objective justification to refer the patient for formal gold-standard testing. This rapid, non-invasive triage approach could help optimize clinical referral pathways without overclaiming diagnostic sensitivity.

The DM group frequently showed globally elevated and comparatively compact temperature distributions. Direct outputs included DM in 7 of 19 participants (36.8%), borderline healthy/suspected DM or controlled DM in 3 of 19 (15.8%), DM suspected PAD in 3 of 19 (15.8%), and PAD_DM in 5 of 19 (26.3%). Because microcirculatory flow, inflammatory biomarkers, and autonomic nerve function were not directly measured in this study, the exact biological drivers of this thermal pattern remain highly speculative. While neuropathic vasomotor dysfunction, specifically autonomic sympathectomy leading to passive arteriovenous shunting, is a well-documented cause of hyperthermal plantar patterns in diabetes, this mechanism cannot be verified in our cohort due to the absence of direct neurological examinations. Consequently, neuropathic thermoregulation and microvascular endothelial dysfunction must be interpreted strictly as unverified pathophysiological hypotheses rather than direct clinical explanations.

The combined PAD_DM cohort most frequently showed mixed, transitional, and highly heterogeneous thermal behavior. Fifteen of 20 participants (75.0%) were assigned directly to PAD_DM, while 5 of 20 (25.0%) were distributed among DM suspected PAD, DM, PAD, and borderline healthy/suspected PAD. Because several patients in this cohort possessed chronic, stable trophic, localized inflammatory cascades, and ischemic necrosis may have directly altered the plantar skin temperatures, contributing to the elevated or atypical thermal profiles. Given the observational nature of this pilot study, it is impossible to isolate or determine whether these thermal abnormalities reflect systemic diabetes-related microcirculatory changes, unmeasured autonomic neuropathy, macrovascular PAD-related ischemia, localized tissue inflammation, or a complex combination of these factors.

A key strength of the present approach is that it treats the foot thermal image as a distributional object. Mean temperature captures the global thermal level, distribution width captures intra-foot thermal heterogeneity, and bilateral asymmetry captures side-to-side imbalance. These parameters provide complementary information. A single mean temperature may miss localized cold or warm regions, while visual inspection alone may overlook subtle distributional shifts. By combining histograms, KDE curves, and rule-based classification, the analysis offers an interpretable bridge between qualitative thermographic assessment and quantitative thermal phenotyping [21].

The rule-based model should be interpreted within the appropriate methodological frame. It is a cohort-derived, patient-level representation of recurring and transitional thermal states, not a fitted group-separation model. The output names were retained because they are concise and intuitive to clinicians reading the decision tree, but they remain thermal-pattern labels and do not independently establish PAD, DM, combined disease, or metabolic control. Sensitivity, specificity, balanced accuracy, macro-F1 score, or cross-validated performance would imply a validation claim that the present design does not provide.

### Limitations of the Study

Several limitations regarding selection bias, confounding, and clinical diagnostics must be noted. First, the small, single-center limits statistical power. Second, the healthy controls were recruited as a convenience sample of younger hospital personnel and were not age- or demographic-matched to the older patient cohorts. Third, the DM-only cohort was composed of carotid artery surgery patients with systemic atherosclerosis; however, PAD was excluded in these patients with clinical examination, ABI and DUS, still representing valuable cohort for thermal pattern in overall DM patients. Furthermore, while acute infections and systemic fevers were excluded, the presence of stable chronic ulcers, dry gangrene, or mild localized edema in some patients might have introduced localized thermal artifacts, however it did not influence overall thermal pattern assessment in these patients.

Another limitation was that the classification rules and temperature boundaries were derived from and evaluated on the same cohort without independent external validation. We did not report diagnostic performance metrics, such as sensitivity, specificity, positive predictive values, or ROC curves. Because no objective neurological testing was performed to assess neuropathy, the confounding effects of neuropathic vasomotor changes cannot be isolated. We did not assess the reproducibility of our manual ROI extraction or inter-/intra-observer measurement reliability. Additionally, the study relied on a single resting measurement rather than dynamic or longitudinal tracking, and patient-level statistical comparisons were limited. Finally, because our descriptive rule-based model and its temperature boundaries were derived from and evaluated on the same dataset without internal or external cross-validation, the classification categories and thresholds may have been defined post hoc and retrospectively tailored to this specific sample. Consequently, the clinical usefulness, diagnostic accuracy, and screening viability of this method for primary healthcare cannot yet be established. Significant further research, specifically prospective, large-scale, multicenter diagnostic accuracy trials using age-matched primary care cohorts, is required before any screening or referral-support utility can be validated.

## 5. Conclusions

This exploratory, cross-sectional study demonstrated that whole-foot plantar temperature distributions can descriptively characterize differences among healthy individuals, patients with isolated DM, and those with combined PAD and DM. While healthy controls generally demonstrated symmetric thermal footprints, the DM cohort frequently exhibited globally elevated and compact temperature-distribution profiles, while mixed asymmetric patterns were common in participants with combined PAD and DM. In contrast, the isolated PAD cohort was characterized by pronounced thermal heterogeneity. No consistent “isolated PAD” thermal pattern could be established using our rule-based model, which is consistent with the highly variable clinical presentation, disease severity, and anatomical laterality of macrovascular arterial disease.

Because of the exploratory nature and small sample size of this study, our cohort-derived model and its associated temperature boundaries must be interpreted strictly as a transparent, descriptive framework of the observed sample rather than a validated diagnostic test. The present data do not support claims regarding the early diagnosis of vascular disease and the identification of diabetic neuropathy; however, characteristic patterns have been observed in these patients that should be validated in future structured studies. Independent, prospective validation using age- and disease severity-matched cohorts is required to determine the reproducibility and potential clinical utility of whole-foot thermal profiling.

## Figures and Tables

**Table 1:** Demographic and baseline clinical characteristics of the study cohorts.

Characteristics	Healthy Controls (*n* = 21)	PAD ^1^ (*n* = 13)	DM ^2^ (*n* = 19)	PAD_DM (*n* = 20)
Age (years, mean ± SD)	32.1 ± 8.6	59.5 ± 10.8	70.2 ± 8.2	70.0 ± 5.1
Sex (% Male)	10 (47.6)	10 (76.9)	10 (52.6)	13 (65.0)
BMI ^3^ (kg/m^2^, mean ± SD)	25.2 ± 4.4	24.4 ± 3.4	28.2 ± 3.9	26.1 ± 3.4
Active Smoking (*n*, %)	14 (66.7)	8 (61.5)	10 (52.6)	9 (45.0)
Hypertension (*n*, %)	1 (4.8)	13 (100.0)	19 (100.0)	20 (100.0)
Dyslipidemia (*n*, %)	0 (0.0)	8 (61.5)	14 (73.7)	16 (80.0)
Duration of DM (years, mean ± SD)	/	/	4.5 ± 2.7	3.1 ± 1.8

^1^ Peripheral arterial disease; ^2^ Diabetes mellitus; ^3^ Body mass index.

**Table 2:** The main variables used in the bilateral representation.

Variable	Variable Description
μ_R ^1^	Mean temperature of the right lower extremity
μ_L ^2^	Mean temperature of the left lower extremity
W_R ^3^	Temperature-distribution width of the right lower extremity
W_L ^4^	Temperature-distribution width of the left lower extremity
Δμ ^5^	Absolute bilateral difference in mean temperature

^1^ mean temperature of the right foot; ^2^ mean temperatures of the left foot; ^3^ temperature distribution width (histogram width) of the right foot; ^4^ temperature distribution width (histogram width) of the left foot; ^5^ absolute differences between the mean temperatures of the right and left feet.

**Table 3:** Vascular and clinical severity characteristics of the PAD and PAD_DM cohorts.

Clinical/Angiographic Parameter	PAD (*n* = 13)	PAD_DM (*n* = 20)
**Mean Ankle-Brachial Index (±SD)**	0.5 ± 0.1	0.5 ± 0.1
**Clinical Severity (Rutherford Category)**		
Category 1–3 (*n*, %)	10 (76.9)	13 (65.0)
Category 4 (*n*, %)	2 (15.4)	2 (10.0)
Category 5–6 (*n*, %)	1 (7.7)	5 (25.0)
**Laterality of Hemodynamically Significant Lesions**		
Unilateral Disease (*n*, %)	4 (30.8)	8 (40.0)
Bilateral Disease (*n*, %)	9 (69.2)	12 (60.0)
**Anatomical Distribution of Lesions**		
Aortoiliac segment (*n*, %)	5 (38.5)	7 (35.0)
Femoropopliteal segment (*n*, %)	8 (61.5)	13 (65.0)
Crural segment (*n*, %)	0 (0.0)	0 (0.0)
**Patent Crural Vessels**		
0–1 Patent Vessel (*n*, %)	2 (15.4)	2 (10.0)
2–3 Patent Vessels (*n*, %)	11 (84.6)	18 (90.0)

**Table 4:** Distribution of original rule-based output categories within each clinically defined cohort, presented as *n* (% within cohort).

Clinical Cohort	Rule-Based Output Categories, *n* (% Within Cohort)
Healthy (*n* = 21)	Healthy: 11 (52.4%); borderline healthy/suspected PAD: 4 (19.0%); borderline healthy/suspected DM or controlled DM: 4 (19.0%); PAD: 1 (4.8%); DM: 1 (4.8%).
PAD (*n* = 13)	PAD_DM: 6 (46.2%); PAD and suspected DM: 2 (15.4%); borderline healthy/suspected DM or controlled DM: 2 (15.4%); DM suspected PAD: 1 (7.7%); DM: 1 (7.7%); healthy: 1 (7.7%); PAD: 0 (0.0%).
DM (*n* = 19)	DM: 7 (36.8%); borderline healthy/suspected DM or controlled DM: 3 (15.8%); DM suspected PAD: 3 (15.8%); PAD_DM: 5 (26.3%); borderline healthy/suspected PAD: 1 (5.3%).
PAD_DM (*n* = 20)	PAD_DM: 15 (75.0%); DM suspected PAD: 1 (5.0%); DM: 2 (10.0%); PAD: 1 (5.0%); borderline healthy/suspected PAD: 1 (5.0%).

## Data Availability

The original contributions presented in this study are included in the article and Supplementary Materials. Further inquiries can be directed to the corresponding authors.

## Supplementary Materials

The following supporting information can be downloaded at: https://www.mdpi.com/article/10.3390/medsci14040487/s1, S1: Overview of the classification framework; S2: Derivation of the cohort-derived descriptive boundaries; Table S1: Cohort-derived descriptive band boundaries used by the rule-based thermal-pattern model; S3: Thermal-pattern output classes; Table S2: Original output classes returned by the rule-based model; S4: Rule-based classification hierarchy; S5: Patient-level descriptive summaries; Table S3: Patient-level right/left thermal-feature summaries by clinical cohort, reported as median [interquartile range]; Table S4: Cross-tabulation of clinically defined cohorts and original rule-based output codes, shown as participant counts; S6: Participant-level rule-based outputs.

## Abbreviations

The following abbreviations are used in this manuscript:

ABI	Ankle-brachial index
CSV	Comma-Separated Values
DFU	Diabetic foot ulceration
DUS	Duplex ultrasound
DM	Diabetes mellitus
KDE	Kernel density estimation
MDCT	Multidetector computed tomography
PAD	Peripheral arterial disease
ROI	Region-of-interest
