# Supplementary material for: See the (In)Visible: Infrared Thermography for Characterizing Thermal Patterns Associated with Peripheral Arterial Disease and Diabetes Mellitus
Source: Med Sci (Basel). 2026 Aug 16;14(4):487. doi: 10.3390/medsci14040487 (PMC13515561; doi:10.3390/medsci14040487)
Supplement: Supplementary file 1 [file medsci-14-00487-s001.zip › medsci-4454731-supplementary.pdf]

Supplementary Information

Cohort-Derived Rule-Based Thermal-Pattern Logic for Bilateral Plantar Infrared Thermography

S1. Overview of the classification framework

A deterministic, rule-based thermal phenotyping model was developed to summarize paired right- and left-foot infrared temperature distributions as clinically interpretable thermal patterns. The model operates at the participant level and integrates absolute thermal level, bilateral mean-temperature asymmetry, and intra-foot trimmed distribution width. Right and left feet are processed separately and retained as one paired bilateral profile; pixels from different participants are not pooled for classification.

The descriptive boundaries were empirically derived from the present study cohort by examining the distribution of thermal features across clinically defined healthy, peripheral arterial disease (PAD), diabetes mellitus (DM), and combined PAD/DM groups. The model should therefore be interpreted as a transparent, cohort-calibrated thermal phenotyping framework rather than as a stand-alone diagnostic test. Its outputs do not represent independent clinical diagnoses or validated measures of diagnostic performance.

The rules are applied sequentially according to a predefined priority hierarchy. Once a case satisfies a rule, the corresponding class is assigned and later rules are not evaluated for that case. This prevents ambiguous class assignment in cases where multiple thermal conditions overlap.

S2. Derivation of the cohort-derived descriptive boundaries

The boundaries used in the rule-based model were developed through exploratory inspection of the present dataset. They were not prespecified clinical diagnostic cut-offs, were not adopted from clinical practice guidelines, and were not optimized using receiver-operating-characteristic analysis or another procedure intended to maximize classification accuracy.

After percentile trimming and patient-level feature extraction, mean temperature, bilateral mean-temperature difference, and temperature-distribution width were examined through patient-level histograms, individual bilateral KDE profiles, and two-dimensional feature-space plots. KDE curves were calculated separately for the right and left foot of each participant. The manuscript presents one representative participant from each basic clinical group; no group-level pooled pixel distribution was used.

The boundaries at 27 °C, 32 °C, and 33 °C therefore represent descriptive temperature bands observed in the present cohort. The bilateral-difference boundaries at 0.25 °C, 0.40 °C, and 0.50 °C describe

progressively greater right-left separation within those bands. The reference width W_ref = 5.0 °C distinguishes relatively broad from comparatively compact temperature distributions. For low-temperature asymmetric cases, the width condition was relaxed by pad_band = 0.8 °C, giving a relaxed boundary of 4.2 °C. This branch was introduced because low-temperature PAD-compatible patterns could retain clinically relevant asymmetry while showing mildly reduced rather than fully preserved distribution width.

The healthy cohort served as a descriptive reference for the range of thermal variability observed in participants without clinically diagnosed PAD or DM. It was not assumed that healthy distributions would be uniform. Borderline and transitional outputs were retained because the method was designed to describe the thermal continuum. Their names are intentionally intuitive for clinical readers, but they remain thermal-pattern labels and should not be interpreted as independent diagnoses or direct evidence of glycemic control.

The thresholds were selected to provide a simple and transparent description of recurring and transitional thermal patterns. They should not be interpreted as validated diagnostic thresholds, and no sensitivity, specificity, predictive value, or independent diagnostic performance is inferred from their application to the same cohort from which they were derived. No internal cross-validation was performed because the rule system was not used to estimate predictive performance. External validation and prospective recalibration are required before these boundaries can be considered for screening-oriented clinical research.

Table S1. Cohort-derived descriptive band boundaries used by the rule-based thermal-pattern model.

Parameter	Value	Interpretation
T_low	27 °C	Lower boundary of the normothermal/intermediate range
T_high	32 °C	Upper boundary of the normothermal/intermediate range
T_very-high	33 °C	High-priority hyperthermal branch
Δμ_small	0.5 °C	Maximum inter-foot difference for thermal symmetry
Δμ_very-small	0.25 °C	Very small asymmetry in high-temperature cases
Δμ_moderate	0.25–0.40 °C	Mild asymmetry in high-temperature cases
W_ref	5.0 °C	Reference distribution width
pad_band	0.8 °C	Relaxation band for PAD-related width assessment
W_loose	W_ref − pad_band = 4.2 °C	Relaxed width threshold
W_very-low	0.5 °C	Extremely narrow thermal width

S3. Thermal-pattern output classes

The model returns a numerical code and a corresponding text label. The original labels were retained because they provide an intuitive clinical reading of the rule hierarchy. They summarize the observed thermal pattern and do not independently establish a clinical diagnosis, disease severity, or glycemic control. In particular, the phrase "suspected DM or controlled DM" describes a compact, relatively symmetric thermal presentation within the intermediate-temperature band; it is not an HbA1c-based assessment. Codes 4 and 7 both represent PAD + DM patterns reached through different thermal branches.

Table S2. Original output classes returned by the rule-based model.

Code	Assigned class
0	Unknown / unclassified
1	Healthy
2	Borderline healthy / suspected PAD
3	Borderline healthy / suspected DM or controlled DM
4	PAD + DM
5	DM
6	PAD
7	PAD + DM
8	DM suspected PAD
9	PAD and suspected DM

S4. Rule-based classification hierarchy

The classification rules are listed below in the same priority order in which they are applied. For each participant, the first satisfied rule determines the final assigned class. The labels are clinically intuitive names for thermal patterns, not stand-alone diagnoses.

Rule 1. Healthy thermal pattern

A case is classified as Healthy when both feet show mean temperatures of at least 27 °C, bilateral thermal asymmetry is small, and both feet show preserved thermal distribution width.

- $\mu\_R \geq 27$ and $\mu\_L \geq 27$
- $\Delta\mu \leq 0.5$
- $W\_R \geq W\_ref$ and $W\_L \geq W\_ref$

Code = 1; Class = Healthy

Rule 2. Borderline healthy / suspected PAD

A case is classified as Borderline healthy / suspected PAD when both feet are within the 27–32 °C interval, inter-foot asymmetry is increased, and thermal width is preserved in both feet.

- $27 \le \mu\_R \le 32$ and $27 \le \mu\_L \le 32$
- $\Delta\mu > 0.5$
- $W\_R \ge W\_ref$ and $W\_L \ge W\_ref$

Code = 2; Class = Borderline healthy / suspected PAD

Rule 3. Borderline healthy / suspected DM or controlled DM

A case is classified as borderline healthy / suspected DM or controlled DM when both feet are within the 27–32 °C interval, inter-foot asymmetry is small, and at least one foot has reduced distribution width. This label describes a thermal presentation and is not a direct assessment of glycemic control.

- $27 \le \mu\_R \le 32$ and $27 \le \mu\_L \le 32$
- $\Delta\mu \le 0.5$
- $W\_R < W\_ref$ or $W\_L < W\_ref$

Code = 3; Class = Borderline healthy / suspected DM or controlled DM

Rule 4. PAD and suspected DM

A case is classified as PAD and suspected DM when both feet are within the 27–32 °C interval, inter-foot asymmetry is increased, and at least one foot has reduced width.

- $27 \le \mu\_R \le 32$ and $27 \le \mu\_L \le 32$
- $\Delta\mu > 0.5$
- $W\_R < W\_ref$ or $W\_L < W\_ref$

Code = 9; Class = PAD and suspected DM

Rule 5. DM in the high-temperature branch

If at least one foot exceeds 33 °C and the bilateral difference is very small, the case is classified as DM.

- $\mu\_R > 33$ or $\mu\_L > 33$
- $\Delta\mu \le 0.25$

Code = 5; Class = DM

Rule 6. DM suspected PAD in the high-temperature branch

If at least one foot exceeds 33 °C and the bilateral difference is mild but not negligible, the case is classified as DM suspected PAD.

- $\mu\_R > 33$ or $\mu\_L > 33$
- $0.25 < \Delta\mu \leq 0.40$

Code = 8; Class = DM suspected PAD

Rule 7. PAD + DM in the high-temperature branch

If at least one foot exceeds 33 °C and the bilateral difference is larger than 0.40 °C, the case is classified as PAD + DM.

- $\mu\_R > 33$ or $\mu\_L > 33$
- $\Delta\mu > 0.40$

Code = 4; Class = PAD + DM

Rule 8. PAD_DM in the >32 °C branch

If the high-priority >33 °C branch is not triggered, cases with at least one foot above 32 °C, increased asymmetry, and reduced width in at least one foot are classified as PAD_DM.

- $\mu\_R > 32$ or $\mu\_L > 32$
- $\Delta\mu > 0.5$
- $W\_R < W\_ref$ or $W\_L < W\_ref$

Code = 4; Class = PAD_DM

Rule 9. DM in the >32 °C branch

If the high-priority >33 °C branch is not triggered, cases with at least one foot above 32 °C, small asymmetry, and reduced width in at least one foot are classified as DM.

- $\mu\_R > 32$ or $\mu\_L > 32$
- $\Delta\mu \leq 0.5$
- $W\_R < W\_ref$ or $W\_L < W\_ref$

Code = 5; Class = DM

Rule 10. PAD_DM in the cross-band asymmetry branch

If one foot is clearly warmer while the other is below 27 °C, asymmetry is strong, and at least one foot has extremely narrow width, the case is classified as PAD_DM.

- $(\mu_R > 28$ and $\mu_L < 27)$ or $(\mu_L > 28$ and $\mu_R < 27)$
- $\Delta\mu > 0.5$
- $\min(W_R, W_L) < 0.5$

Code = 4; Class = PAD_DM

Rule 11. PAD in the cross-band asymmetry branch

If the cross-band condition is present with strong asymmetry but without extremely narrow width, the case is classified as PAD.

- $(\mu_R > 28$ and $\mu_L < 27)$ or $(\mu_L > 28$ and $\mu_R < 27)$
- $\Delta\mu > 0.5$

Code = 6; Class = PAD

Rule 12. PAD in the low-temperature branch

If at least one foot is below 27 °C, bilateral asymmetry is increased, and both feet retain sufficient width under the relaxed PAD threshold, the case is classified as PAD.

- $\mu_R < 27$ or $\mu_L < 27$
- $\Delta\mu > 0.5$
- $W_R \geq W_{ref} - pad\_band$ and $W_L \geq W_{ref} - pad\_band$

Code = 6; Class = PAD

Rule 13. PAD + DM in the low-temperature branch

If at least one foot is below 27 °C, bilateral asymmetry is increased, and at least one foot has width below the relaxed threshold, the case is classified as PAD_DM.

- $\mu_R < 27$ or $\mu_L < 27$
- $\Delta\mu > 0.5$
- $W_R < W_{ref} - pad\_band$ or $W_L < W_{ref} - pad\_band$

Code = 7; Class = PAD + DM

S5. Patient-level descriptive summaries

The participant was the unit of analysis. Table S3 reports median [interquartile range] values for right- and left-foot mean temperature, median temperature, distribution width, and bilateral asymmetry. These summaries provide cohort context while preserving the separate right/left measurements; they are not used to replace the participant-level rule-based interpretation.

Table S3. Patient-level right/left thermal-feature summaries by clinical cohort, reported as median [interquartile range].

Group	μ_R	μ_L	Median_R	Median_L	W_R	W_L	Δμ
**Healthy**	30.44 [29.23-31.06]	30.32 [29.65-31.16]	30.50 [29.30-31.20]	30.40 [29.50-31.40]	5.80 [5.00-6.70]	5.80 [5.10-6.20]	0.22 [0.15-0.49]
**PAD**	32.93 [30.77-34.28]	33.07 [31.68-34.22]	33.00 [30.90-34.50]	33.20 [31.70-34.30]	3.80 [3.50-4.70]	4.40 [3.00-5.30]	0.87 [0.36-1.44]
**DM**	34.36 [32.21-35.01]	34.67 [32.40-35.05]	34.40 [32.25-35.05]	34.70 [32.50-35.10]	3.70 [3.45-4.35]	3.70 [3.45-4.40]	0.27 [0.13-0.44]
**PAD/DM**	33.98 [32.60-35.63]	33.82 [31.90-35.52]	33.95 [32.70-35.60]	33.95 [32.20-35.67]	4.00 [3.60-4.92]	4.30 [3.55-5.03]	1.14 [0.56-1.73]

Table S4. Cross-tabulation of clinically defined cohorts and original rule-based output codes, shown as participant counts.

Clinical cohort	1	2	3	4/7	5	6	8	9
**Healthy**	11	4	4	0	1	1	0	0
**PAD**	1	0	2	6	1	0	1	2
**DM**	0	1	3	5	7	0	3	0
**PAD/DM**	0	1	0	15	2	1	1	0

Code labels: 1, Healthy; 2, Borderline healthy / suspected PAD; 3, Borderline healthy / suspected DM or controlled DM; 4/7, PAD + DM; 5, DM; 6, PAD; 8, DM suspected PAD; 9, PAD and suspected DM. This table is a descriptive cross-tabulation, not an independently validated confusion matrix.

S6. Participant-level rule-based outputs

=== Classification results ===

--- Evaluating group: Healthy ---

Healthy sample 1: code=1, desc=Healthy, diag={'R_stats': {'mean': 32.24461665292663, 'median': 32.3, 'width': 5.5000000000000036}, 'L_stats': {'mean': 32.397002158445915, 'median': 32.5, 'width': 5.399999999999999}, 'diff_mean': 0.15238550551928398, 'width_ref': 5, 'pad_band': 0.8, 'R_label':

'desna25.csv', 'L_label': 'leva25.csv'}

Healthy sample 2: code=1, desc=Healthy, diag={'R_stats': {'mean': 31.055557812420655, 'median': 31.3, 'width': 5.099999999999998}, 'L_stats': {'mean': 31.46349145565076, 'median': 31.5, 'width': 5.099999999999998}, 'diff_mean': 0.4079336432301055, 'width_ref': 5, 'pad_band': 0.8, 'R_label': 'desna26.csv', 'L_label': 'leva26.csv'}

Healthy sample 3: code=2, desc=Borderline healthy / Suspected PAD, diag={'R_stats': {'mean': 29.618097052336076, 'median': 29.6, 'width': 7.200000000000003}, 'L_stats': {'mean': 30.38636407254582, 'median': 30.4, 'width': 6.600000000000001}, 'diff_mean': 0.7682670202097448, 'width_ref': 5, 'pad_band': 0.8, 'R_label': 'desna27.csv', 'L_label': 'leva27.csv'}

Healthy sample 4: code=3, desc=Borderline healthy / Suspected DM or controlled DM, diag={'R_stats': {'mean': 29.896057082452426, 'median': 29.9, 'width': 4.399999999999999}, 'L_stats': {'mean': 29.652877215629807, 'median': 29.5, 'width': 5.800000000000001}, 'diff_mean': 0.2431798668226186, 'width_ref': 5, 'pad_band': 0.8, 'R_label': 'desna28.csv', 'L_label': 'leva28.csv'}

Healthy sample 5: code=1, desc=Healthy, diag={'R_stats': {'mean': 30.95080588063804, 'median': 31.0, 'width': 6.700000000000003}, 'L_stats': {'mean': 30.67375944124096, 'median': 30.6, 'width': 6.0000000000000036}, 'diff_mean': 0.27704643939707907, 'width_ref': 5, 'pad_band': 0.8, 'R_label': 'desna32.csv', 'L_label': 'leva32.csv'}

Healthy sample 6: code=3, desc=Borderline healthy / Suspected DM or controlled DM, diag={'R_stats': {'mean': 30.5431509206677, 'median': 30.7, 'width': 5.0}, 'L_stats': {'mean': 30.323013365735115, 'median': 30.5, 'width': 3.8000000000000007}, 'diff_mean': 0.22013755493258458, 'width_ref': 5, 'pad_band': 0.8, 'R_label': 'desna33.csv', 'L_label': 'leva33.csv'}

Healthy sample 7: code=1, desc=Healthy, diag={'R_stats': {'mean': 31.79000996381562, 'median': 31.9, 'width': 8.500000000000004}, 'L_stats': {'mean': 31.64386617100372, 'median': 31.7, 'width': 8.699999999999996}, 'diff_mean': 0.14614379281190182, 'width_ref': 5, 'pad_band': 0.8, 'R_label': 'desna34.csv', 'L_label': 'leva34.csv'}

Healthy sample 8: code=1, desc=Healthy, diag={'R_stats': {'mean': 32.07519032224424, 'median':

32.2, 'width': 6.100000000000001}, 'L_stats': {'mean': 32.1832419161051, 'median': 32.3, 'width': 6.300000000000001}, 'diff_mean': 0.10805159386086416, 'width_ref': 5, 'pad_band': 0.8, 'R_label': 'desna36.csv', 'L_label': 'leva36.csv'}

Healthy sample 9: code=1, desc=Healthy, diag={'R_stats': {'mean': 28.645063602790316, 'median': 28.5, 'width': 9.099999999999998}, 'L_stats': {'mean': 28.66226257914029, 'median': 28.5, 'width': 8.799999999999997}, 'diff_mean': 0.017198976349973805, 'width_ref': 5, 'pad_band': 0.8, 'R_label': 'desna37.csv', 'L_label': 'leva37.csv'}

Healthy sample 10: code=3, desc=Borderline healthy / Suspected DM or controlled DM, diag={'R_stats': {'mean': 30.56385147247119, 'median': 30.8, 'width': 4.799999999999997}, 'L_stats': {'mean': 30.730758888556917, 'median': 30.9, 'width': 4.699999999999999}, 'diff_mean': 0.1669074160857278, 'width_ref': 5, 'pad_band': 0.8, 'R_label': 'desna38.csv', 'L_label': 'leva38.csv'}

Healthy sample 11: code=1, desc=Healthy, diag={'R_stats': {'mean': 31.204063725490197, 'median': 31.2, 'width': 6.800000000000004}, 'L_stats': {'mean': 31.159852193108744, 'median': 31.4, 'width': 5.800000000000001}, 'diff_mean': 0.04421153238145337, 'width_ref': 5, 'pad_band': 0.8, 'R_label': 'desna39.csv', 'L_label': 'leva39.csv'}

Healthy sample 12: code=2, desc=Borderline healthy / Suspected PAD, diag={'R_stats': {'mean': 29.992994481954963, 'median': 30.4, 'width': 7.399999999999999}, 'L_stats': {'mean': 30.827957327736968, 'median': 31.0, 'width': 5.699999999999999}, 'diff_mean': 0.8349628457820053, 'width_ref': 5, 'pad_band': 0.8, 'R_label': 'desna43.csv', 'L_label': 'leva43.csv'}

Healthy sample 13: code=5, desc=DM, diag={'R_stats': {'mean': 32.59749408983451, 'median': 32.7, 'width': 4.700000000000003}, 'L_stats': {'mean': 32.5229094736842, 'median': 32.5, 'width': 4.899999999999999}, 'diff_mean': 0.07458461615031098, 'width_ref': 5, 'pad_band': 0.8, 'R_label': 'desna44.csv', 'L_label': 'leva44.csv'}

Healthy sample 14: code=6, desc=PAD, diag={'R_stats': {'mean': 26.691378190255225, 'median': 26.7, 'width': 4.300000000000001}, 'L_stats': {'mean': 27.445401019246944, 'median': 27.4, 'width': 5.100000000000001}, 'diff_mean': 0.7540228289917188, 'width_ref': 5, 'pad_band': 0.8, 'R_label': 'desna45.csv', 'L_label': 'leva45.csv'}

Healthy sample 15: code=1, desc=Healthy, diag={'R_stats': {'mean': 29.232794073821786, 'median': 29.3, 'width': 5.399999999999999}, 'L_stats': {'mean': 29.666435459313142, 'median': 29.7, 'width': 5.400000000000002}, 'diff_mean': 0.4336413854913559, 'width_ref': 5, 'pad_band': 0.8, 'R_label': 'desna46.csv', 'L_label': 'leva46.csv'}

Healthy sample 16: code=1, desc=Healthy, diag={'R_stats': {'mean': 27.392371926015613, 'median': 27.6, 'width': 6.600000000000001}, 'L_stats': {'mean': 27.44924051661116, 'median': 27.6, 'width': 6.0}, 'diff_mean': 0.0568685905955455, 'width_ref': 5, 'pad_band': 0.8, 'R_label': 'desna48.csv', 'L_label': 'leva48.csv'}

Healthy sample 17: code=2, desc=Borderline healthy / Suspected PAD, diag={'R_stats': {'mean': 30.44149152327132, 'median': 30.5, 'width': 5.4999999999999964}, 'L_stats': {'mean': 29.8840099610079, 'median': 30.2, 'width': 6.199999999999999}, 'diff_mean': 0.557481562263419, 'width_ref': 5, 'pad_band': 0.8, 'R_label': 'desna49.csv', 'L_label': 'leva49.csv'}

Healthy sample 18: code=1, desc=Healthy, diag={'R_stats': {'mean': 29.637965134060558, 'median': 29.5, 'width': 6.4999999999999964}, 'L_stats': {'mean': 29.804124419441358, 'median': 29.8, 'width': 6.099999999999998}, 'diff_mean': 0.16615928538080027, 'width_ref': 5, 'pad_band': 0.8, 'R_label': 'desna50.csv', 'L_label': 'leva50.csv'}

Healthy sample 19: code=2, desc=Borderline healthy / Suspected PAD, diag={'R_stats': {'mean': 28.3596268869268, 'median': 28.6, 'width': 5.799999999999997}, 'L_stats': {'mean': 27.848733691481197, 'median': 27.8, 'width': 5.900000000000002}, 'diff_mean': 0.510893195445604, 'width_ref': 5, 'pad_band': 0.8, 'R_label': 'desna55.csv', 'L_label': 'leva55.csv'}

Healthy sample 20: code=3, desc=Borderline healthy / Suspected DM or controlled DM, diag={'R_stats': {'mean': 28.895193530801382, 'median': 29.0, 'width': 4.799999999999997}, 'L_stats': {'mean': 29.111339301386693, 'median': 29.3, 'width': 4.899999999999999}, 'diff_mean': 0.21614577058531026, 'width_ref': 5, 'pad_band': 0.8, 'R_label': 'desna104.csv', 'L_label': 'leva104.csv'}

Healthy sample 21: code=1, desc=Healthy, diag={'R_stats': {'mean': 30.540630375392087, 'median':

30.7, 'width': 6.699999999999999}, 'L_stats': {'mean': 30.050889063729343, 'median': 30.0, 'width': 7.700000000000003}, 'diff_mean': 0.48974131166274404, 'width_ref': 5, 'pad_band': 0.8, 'R_label': 'desna118.csv', 'L_label': 'leva118.csv'}

--- Evaluating group: PAD ---

PAD sample 1: code=1, desc=Healthy, diag={'R_stats': {'mean': 28.04339039312679, 'median': 28.4, 'width': 5.899999999999999}, 'L_stats': {'mean': 28.4061774529581, 'median': 28.5, 'width': 7.299999999999997}, 'diff_mean': 0.3627870598313123, 'width_ref': 5, 'pad_band': 0.8, 'R_label': 'desna30.csv', 'L_label': 'leva30.csv'}

PAD sample 2: code=3, desc=Borderline healthy / Suspected DM or controlled DM, diag={'R_stats': {'mean': 28.927098708487087, 'median': 28.9, 'width': 2.8000000000000007}, 'L_stats': {'mean': 28.928880668584736, 'median': 28.9, 'width': 3.0}, 'diff_mean': 0.0017819600976487493, 'width_ref': 5, 'pad_band': 0.8, 'R_label': 'desna56.csv', 'L_label': 'leva56.csv'}

PAD sample 3: code=9, desc=PAD and suspected DM, diag={'R_stats': {'mean': 30.76834132794749, 'median': 30.9, 'width': 4.200000000000003}, 'L_stats': {'mean': 31.6771131862824, 'median': 31.7, 'width': 5.299999999999997}, 'diff_mean': 0.9087718583349123, 'width_ref': 5, 'pad_band': 0.8, 'R_label': 'desna57.csv', 'L_label': 'leva57.csv'}

PAD sample 4: code=4, desc=PAD + DM, diag={'R_stats': {'mean': 32.573939732142854, 'median': 32.7, 'width': 4.699999999999999}, 'L_stats': {'mean': 31.70610748037049, 'median': 31.8, 'width': 5.300000000000004}, 'diff_mean': 0.8678322517723629, 'width_ref': 5, 'pad_band': 0.8, 'R_label': 'desna63.csv', 'L_label': 'leva63.csv'}

PAD sample 5: code=9, desc=PAD and suspected DM, diag={'R_stats': {'mean': 29.261313572226165, 'median': 29.2, 'width': 3.799999999999997}, 'L_stats': {'mean': 31.887786376915585, 'median': 31.9, 'width': 4.299999999999997}, 'diff_mean': 2.6264728046894206, 'width_ref': 5, 'pad_band': 0.8, 'R_label': 'desna64.csv', 'L_label': 'leva64.csv'}

PAD sample 6: code=4, desc=PAD + DM, diag={'R_stats': {'mean': 35.65790043290043, 'median': 35.9, 'width': 5.400000000000006}, 'L_stats': {'mean': 34.21526573095726, 'median': 34.3, 'width':

4.599999999999998}, 'diff_mean': 1.4426347019431702, 'width_ref': 5, 'pad_band': 0.8, 'R_label': 'desna74.csv', 'L_label': 'leva74.csv'}

PAD sample 7: code=4, desc=PAD + DM, diag={'R_stats': {'mean': 33.93582704466653, 'median': 33.9, 'width': 3.5}, 'L_stats': {'mean': 34.77128712871287, 'median': 34.6, 'width': 6.800000000000001}, 'diff_mean': 0.8354600840463391, 'width_ref': 5, 'pad_band': 0.8, 'R_label': 'desna79.csv', 'L_label': 'leva79.csv'}

PAD sample 8: code=3, desc=Borderline healthy / Suspected DM or controlled DM, diag={'R_stats': {'mean': 31.289939898289415, 'median': 31.5, 'width': 3.6000000000000014}, 'L_stats': {'mean': 31.656974413944166, 'median': 31.7, 'width': 2.8000000000000007}, 'diff_mean': 0.3670345156547512, 'width_ref': 5, 'pad_band': 0.8, 'R_label': 'desna90.csv', 'L_label': 'leva90.csv'}

PAD sample 9: code=5, desc=DM, diag={'R_stats': {'mean': 36.39535532379335, 'median': 36.5, 'width': 2.200000000000003}, 'L_stats': {'mean': 36.3062685179806, 'median': 36.4, 'width': 2.700000000000003}, 'diff_mean': 0.08908680581275519, 'width_ref': 5, 'pad_band': 0.8, 'R_label': 'desna105.csv', 'L_label': 'leva105.csv'}

PAD sample 10: code=4, desc=PAD + DM, diag={'R_stats': {'mean': 33.80771195097038, 'median': 34.1, 'width': 5.699999999999999}, 'L_stats': {'mean': 36.04623854999026, 'median': 36.1, 'width': 4.799999999999997}, 'diff_mean': 2.2385265990198775, 'width_ref': 5, 'pad_band': 0.8, 'R_label': 'desna108.csv', 'L_label': 'leva108.csv'}

PAD sample 11: code=8, desc=DM suspected PAD, diag={'R_stats': {'mean': 34.28207719538655, 'median': 34.5, 'width': 4.599999999999998}, 'L_stats': {'mean': 33.928693130324454, 'median': 34.0, 'width': 4.399999999999999}, 'diff_mean': 0.3533840650620945, 'width_ref': 5, 'pad_band': 0.8, 'R_label': 'desna110.csv', 'L_label': 'leva110.csv'}

PAD sample 12: code=4, desc=PAD + DM, diag={'R_stats': {'mean': 32.92936952159679, 'median': 33.0, 'width': 3.8000000000000043}, 'L_stats': {'mean': 33.82420044291085, 'median': 33.9, 'width': 2.6999999999999957}, 'diff_mean': 0.8948309213140604, 'width_ref': 5, 'pad_band': 0.8, 'R_label': 'desna113.csv', 'L_label': 'leva113.csv'}

PAD sample 13: code=4, desc=PAD + DM, diag={'R_stats': {'mean': 35.264796633941096, 'median': 35.4, 'width': 2.799999999999997}, 'L_stats': {'mean': 33.06574147501983, 'median': 33.2, 'width': 3.099999999999998}, 'diff_mean': 2.1990551589212686, 'width_ref': 5, 'pad_band': 0.8, 'R_label': 'desna120.csv', 'L_label': 'leva120.csv'}

--- Evaluating group: PAD_DM ---

PAD_DM sample 1: code=2, desc=Borderline healthy / Suspected PAD, diag={'R_stats': {'mean': 29.566709126329986, 'median': 29.7, 'width': 5.0}, 'L_stats': {'mean': 30.19637091113295, 'median': 30.1, 'width': 5.100000000000001}, 'diff_mean': 0.6296617848029626, 'width_ref': 5, 'pad_band': 0.8, 'R_label': 'desna51.csv', 'L_label': 'leva51.csv'}

PAD_DM sample 2: code=4, desc=PAD + DM, diag={'R_stats': {'mean': 32.42082092726499, 'median': 32.6, 'width': 4.300000000000004}, 'L_stats': {'mean': 33.006257512019225, 'median': 33.2, 'width': 3.6000000000000014}, 'diff_mean': 0.585436584754234, 'width_ref': 5, 'pad_band': 0.8, 'R_label': 'desna60.csv', 'L_label': 'leva60.csv'}

PAD_DM sample 3: code=4, desc=PAD + DM, diag={'R_stats': {'mean': 36.27344784115978, 'median': 36.4, 'width': 3.6000000000000014}, 'L_stats': {'mean': 35.7847181931206, 'median': 35.9, 'width': 4.300000000000004}, 'diff_mean': 0.4887296480391825, 'width_ref': 5, 'pad_band': 0.8, 'R_label': 'desna62.csv', 'L_label': 'leva62.csv'}

PAD_DM sample 4: code=8, desc=DM suspected PAD, diag={'R_stats': {'mean': 35.63875988557967, 'median': 35.6, 'width': 3.6999999999999957}, 'L_stats': {'mean': 35.98165598635379, 'median': 36.0, 'width': 2.8000000000000043}, 'diff_mean': 0.34289610077412647, 'width_ref': 5, 'pad_band': 0.8, 'R_label': 'desna73.csv', 'L_label': 'leva73.csv'}

PAD_DM sample 5: code=4, desc=PAD + DM, diag={'R_stats': {'mean': 34.46850283647126, 'median': 34.4, 'width': 4.599999999999998}, 'L_stats': {'mean': 33.27724004909696, 'median': 33.4, 'width': 3.3999999999999986}, 'diff_mean': 1.1912627873742991, 'width_ref': 5, 'pad_band': 0.8, 'R_label': 'desna76.csv', 'L_label': 'leva76.csv'}

PAD_DM sample 6: code=4, desc=PAD + DM, diag={'R_stats': {'mean': 31.319975887971808, 'median': 31.3, 'width': 3.8999999999999986}, 'L_stats': {'mean': 33.4587270155587, 'median': 33.5, 'width': 3.900000000000002}, 'diff_mean': 2.138751127586893, 'width_ref': 5, 'pad_band': 0.8, 'R_label': 'desna77.csv', 'L_label': 'leva77.csv'}

PAD_DM sample 7: code=4, desc=PAD + DM, diag={'R_stats': {'mean': 32.859677188973, 'median': 32.8, 'width': 5.300000000000001}, 'L_stats': {'mean': 31.763082287308226, 'median': 31.7, 'width': 3.1999999999999993}, 'diff_mean': 1.0965949016647762, 'width_ref': 5, 'pad_band': 0.8, 'R_label': 'desna82.csv', 'L_label': 'leva82.csv'}

PAD_DM sample 8: code=4, desc=PAD + DM, diag={'R_stats': {'mean': 34.72920762176705, 'median': 34.9, 'width': 3.5999999999999943}, 'L_stats': {'mean': 35.422682584269666, 'median': 35.4, 'width': 5.299999999999997}, 'diff_mean': 0.6934749625026129, 'width_ref': 5, 'pad_band': 0.8, 'R_label': 'desna83.csv', 'L_label': 'leva83.csv'}

PAD_DM sample 9: code=4, desc=PAD + DM, diag={'R_stats': {'mean': 34.8802026880555, 'median': 34.8, 'width': 4.0}, 'L_stats': {'mean': 36.070007229853736, 'median': 36.0, 'width': 4.600000000000001}, 'diff_mean': 1.1898045417982388, 'width_ref': 5, 'pad_band': 0.8, 'R_label': 'desna85.csv', 'L_label': 'leva85.csv'}

PAD_DM sample 10: code=4, desc=PAD + DM, diag={'R_stats': {'mean': 34.9728890326755, 'median': 35.1, 'width': 3.1000000000000014}, 'L_stats': {'mean': 34.56041643795513, 'median': 34.8, 'width': 3.6000000000000014}, 'diff_mean': 0.41247259472037, 'width_ref': 5, 'pad_band': 0.8, 'R_label': 'desna93.csv', 'L_label': 'leva93.csv'}

PAD_DM sample 11: code=4, desc=PAD + DM, diag={'R_stats': {'mean': 36.43597889475146, 'median': 36.7, 'width': 7.200000000000003}, 'L_stats': {'mean': 34.05841804838272, 'median': 34.2, 'width': 5.600000000000001}, 'diff_mean': 2.3775608463687377, 'width_ref': 5, 'pad_band': 0.8, 'R_label': 'desna94.csv', 'L_label': 'leva94.csv'}

PAD_DM sample 12: code=5, desc=DM, diag={'R_stats': {'mean': 37.85772537460083, 'median':

37.9, 'width': 3.3999999999999986}, 'L_stats': {'mean': 38.04737771315816, 'median': 38.1, 'width': 3.8000000000000043}, 'diff_mean': 0.18965233855733032, 'width_ref': 5, 'pad_band': 0.8, 'R_label': 'desna95.csv', 'L_label': 'leva95.csv'}

PAD_DM sample 13: code=4, desc=PAD + DM, diag={'R_stats': {'mean': 32.47762528592223, 'median': 32.7, 'width': 4.899999999999999}, 'L_stats': {'mean': 31.80180548039553, 'median': 31.9, 'width': 5.299999999999997}, 'diff_mean': 0.6758198055267002, 'width_ref': 5, 'pad_band': 0.8, 'R_label': 'desna96.csv', 'L_label': 'leva96.csv'}

PAD_DM sample 14: code=4, desc=PAD + DM, diag={'R_stats': {'mean': 33.486771910724, 'median': 33.5, 'width': 3.8999999999999986}, 'L_stats': {'mean': 31.929019718309853, 'median': 32.3, 'width': 5.0}, 'diff_mean': 1.5577521924141493, 'width_ref': 5, 'pad_band': 0.8, 'R_label': 'desna101.csv', 'L_label': 'leva101.csv'}

PAD_DM sample 15: code=4, desc=PAD + DM, diag={'R_stats': {'mean': 32.81680291074044, 'median': 32.9, 'width': 5.199999999999999}, 'L_stats': {'mean': 34.30021466237342, 'median': 34.2, 'width': 3.3999999999999986}, 'diff_mean': 1.4834117516329783, 'width_ref': 5, 'pad_band': 0.8, 'R_label': 'desna107.csv', 'L_label': 'leva107.csv'}

PAD_DM sample 16: code=5, desc=DM, diag={'R_stats': {'mean': 35.62891124810981, 'median': 35.6, 'width': 2.200000000000003}, 'L_stats': {'mean': 35.85696237147233, 'median': 35.9, 'width': 2.3000000000000043}, 'diff_mean': 0.2280511233625191, 'width_ref': 5, 'pad_band': 0.8, 'R_label': 'desna116.csv', 'L_label': 'leva116.csv'}

PAD_DM sample 17: code=6, desc=PAD, diag={'R_stats': {'mean': 30.00246152251645, 'median': 30.1, 'width': 4.0}, 'L_stats': {'mean': 26.348979488478093, 'median': 26.5, 'width': 4.800000000000001}, 'diff_mean': 3.6534820340383583, 'width_ref': 5, 'pad_band': 0.8, 'R_label': 'desna123.csv', 'L_label': 'leva123.csv'}

PAD_DM sample 18: code=4, desc=PAD + DM, diag={'R_stats': {'mean': 32.668845574473906, 'median': 32.7, 'width': 2.6999999999999993}, 'L_stats': {'mean': 31.069113893278228, 'median': 31.2, 'width': 4.600000000000001}, 'diff_mean': 1.5997316811956779, 'width_ref': 5, 'pad_band': 0.8, 'R_label': 'desna125.csv', 'L_label': 'leva125.csv'}

PAD_DM sample 19: code=4, desc=PAD + DM, diag={'R_stats': {'mean': 32.63468283582089, 'median': 33.0, 'width': 4.100000000000001}, 'L_stats': {'mean': 35.43562071866362, 'median': 35.6, 'width': 4.300000000000004}, 'diff_mean': 2.8009378828427316, 'width_ref': 5, 'pad_band': 0.8, 'R_label': 'desna126.csv', 'L_label': 'leva126.csv'}

PAD_DM sample 20: code=4, desc=PAD + DM, diag={'R_stats': {'mean': 36.47585406301824, 'median': 36.6, 'width': 6.700000000000003}, 'L_stats': {'mean': 33.58174210977136, 'median': 33.7, 'width': 5.599999999999998}, 'diff_mean': 2.8941119532468775, 'width_ref': 5, 'pad_band': 0.8, 'R_label': 'desna135.csv', 'L_label': 'leva135.csv'}

--- Evaluating group: DM ---

DM sample 1: code=4, desc=PAD + DM, diag={'R_stats': {'mean': 34.70230586911983, 'median': 34.8, 'width': 3.9000000000000057}, 'L_stats': {'mean': 35.15689915699062, 'median': 35.1, 'width': 3.6000000000000014}, 'diff_mean': 0.4545932878707859, 'width_ref': 5, 'pad_band': 0.8, 'R_label': 'desna40.csv', 'L_label': 'leva40.csv'}

DM sample 2: code=2, desc=Borderline healthy / Suspected PAD, diag={'R_stats': {'mean': 29.385419978802336, 'median': 29.7, 'width': 5.300000000000001}, 'L_stats': {'mean': 29.938713910761155, 'median': 30.1, 'width': 5.5}, 'diff_mean': 0.5532939319588195, 'width_ref': 5, 'pad_band': 0.8, 'R_label': 'desna52.csv', 'L_label': 'leva52.csv'}

DM sample 3: code=3, desc=Borderline healthy / Suspected DM or controlled DM, diag={'R_stats': {'mean': 29.985335227272728, 'median': 30.0, 'width': 3.6999999999999993}, 'L_stats': {'mean': 29.81286679153187, 'median': 29.9, 'width': 4.099999999999998}, 'diff_mean': 0.17246843574085702, 'width_ref': 5, 'pad_band': 0.8, 'R_label': 'desna61.csv', 'L_label': 'leva61.csv'}

DM sample 4: code=4, desc=PAD + DM, diag={'R_stats': {'mean': 34.20259679821296, 'median': 34.3, 'width': 4.199999999999999}, 'L_stats': {'mean': 33.24758714350384, 'median': 33.5, 'width': 4.399999999999999}, 'diff_mean': 0.9550096547091158, 'width_ref': 5, 'pad_band': 0.8, 'R_label': 'desna67.csv', 'L_label': 'leva67.csv'}

DM sample 5: code=5, desc=DM, diag={'R_stats': {'mean': 31.85946496682167, 'median': 31.8, 'width': 4.5}, 'L_stats': {'mean': 32.27995387817087, 'median': 32.3, 'width': 3.8999999999999986}, 'diff_mean': 0.42048891134920297, 'width_ref': 5, 'pad_band': 0.8, 'R_label': 'desna68.csv', 'L_label': 'leva68.csv'}

DM sample 6: code=4, desc=PAD + DM, diag={'R_stats': {'mean': 32.594623395495276, 'median': 32.7, 'width': 3.599999999999998}, 'L_stats': {'mean': 33.30594639099808, 'median': 33.4, 'width': 3.6000000000000014}, 'diff_mean': 0.7113229955028046, 'width_ref': 5, 'pad_band': 0.8, 'R_label': 'desna69.csv', 'L_label': 'leva69.csv'}

DM sample 7: code=3, desc=Borderline healthy / Suspected DM or controlled DM, diag={'R_stats': {'mean': 31.146287449211943, 'median': 31.2, 'width': 3.3999999999999986}, 'L_stats': {'mean': 31.435841893702545, 'median': 31.5, 'width': 3.299999999999997}, 'diff_mean': 0.2895544444906015, 'width_ref': 5, 'pad_band': 0.8, 'R_label': 'desna78.csv', 'L_label': 'leva78.csv'}

DM sample 8: code=8, desc=DM suspected PAD, diag={'R_stats': {'mean': 34.032165470662726, 'median': 34.1, 'width': 2.8999999999999986}, 'L_stats': {'mean': 34.42131125650955, 'median': 34.4, 'width': 3.6999999999999957}, 'diff_mean': 0.38914578584682147, 'width_ref': 5, 'pad_band': 0.8, 'R_label': 'desna80.csv', 'L_label': 'leva80.csv'}

DM sample 9: code=3, desc=Borderline healthy / Suspected DM or controlled DM, diag={'R_stats': {'mean': 31.62196781509882, 'median': 31.7, 'width': 5.799999999999997}, 'L_stats': {'mean': 31.59584978070176, 'median': 31.5, 'width': 4.899999999999999}, 'diff_mean': 0.026118034397061507, 'width_ref': 5, 'pad_band': 0.8, 'R_label': 'desna86.csv', 'L_label': 'leva86.csv'}

DM sample 10: code=8, desc=DM suspected PAD, diag={'R_stats': {'mean': 35.76063807220737, 'median': 35.9, 'width': 4.600000000000001}, 'L_stats': {'mean': 36.02981446793328, 'median': 36.1, 'width': 4.399999999999999}, 'diff_mean': 0.269176395725907, 'width_ref': 5, 'pad_band': 0.8, 'R_label': 'desna91.csv', 'L_label': 'leva91.csv'}

DM sample 11: code=5, desc=DM, diag={'R_stats': {'mean': 35.04805843583393, 'median': 35.1, 'width': 3.6000000000000014}, 'L_stats': {'mean': 34.980364476386036, 'median': 35.0, 'width': 5.199999999999996}, 'diff_mean': 0.0676939594478938, 'width_ref': 5, 'pad_band': 0.8, 'R_label':

'desna92.csv', 'L_label': 'leva92.csv'}

DM sample 12: code=5, desc=DM, diag={'R_stats': {'mean': 34.767708492926815, 'median': 34.8, 'width': 3.5999999999999943}, 'L_stats': {'mean': 34.66751923587158, 'median': 34.8, 'width': 3.0}, 'diff_mean': 0.1001892570552343, 'width_ref': 5, 'pad_band': 0.8, 'R_label': 'desna97.csv', 'L_label': 'leva97.csv'}

DM sample 13: code=8, desc=DM suspected PAD, diag={'R_stats': {'mean': 35.21776120107644, 'median': 35.4, 'width': 2.700000000000003}, 'L_stats': {'mean': 34.96746921521778, 'median': 35.1, 'width': 3.5}, 'diff_mean': 0.2502919858586594, 'width_ref': 5, 'pad_band': 0.8, 'R_label': 'desna99.csv', 'L_label': 'leva99.csv'}

DM sample 14: code=5, desc=DM, diag={'R_stats': {'mean': 34.643190060443246, 'median': 34.9, 'width': 4.5}, 'L_stats': {'mean': 34.87273455836053, 'median': 35.0, 'width': 4.899999999999999}, 'diff_mean': 0.22954449791728138, 'width_ref': 5, 'pad_band': 0.8, 'R_label': 'desna106.csv', 'L_label': 'leva106.csv'}

DM sample 15: code=5, desc=DM, diag={'R_stats': {'mean': 32.56341762965443, 'median': 32.7, 'width': 4.199999999999999}, 'L_stats': {'mean': 32.521093361339894, 'median': 32.7, 'width': 4.0}, 'diff_mean': 0.042324268314537505, 'width_ref': 5, 'pad_band': 0.8, 'R_label': 'desna111.csv', 'L_label': 'leva111.csv'}

DM sample 16: code=4, desc=PAD + DM, diag={'R_stats': {'mean': 35.86567519635166, 'median': 35.8, 'width': 3.200000000000003}, 'L_stats': {'mean': 35.3751257123701, 'median': 35.4, 'width': 3.1000000000000014}, 'diff_mean': 0.4905494839815603, 'width_ref': 5, 'pad_band': 0.8, 'R_label': 'desna112.csv', 'L_label': 'leva112.csv'}

DM sample 17: code=5, desc=DM, diag={'R_stats': {'mean': 36.439911775987724, 'median': 36.4, 'width': 3.1000000000000014}, 'L_stats': {'mean': 36.4146876141761, 'median': 36.4, 'width': 3.4000000000000057}, 'diff_mean': 0.02522416181162157, 'width_ref': 5, 'pad_band': 0.8, 'R_label': 'desna117.csv', 'L_label': 'leva117.csv'}

DM sample 18: code=4, desc=PAD + DM, diag={'R_stats': {'mean': 34.35570434477516, 'median':

34.4, 'width': 3.5}, 'L_stats': {'mean': 34.777780284231895, 'median': 34.7, 'width': 3.0}, 'diff_mean': 0.4220759394567324, 'width_ref': 5, 'pad_band': 0.8, 'R_label': 'desna119.csv', 'L_label': 'leva119.csv'}

DM sample 19: code=5, desc=DM, diag={'R_stats': {'mean': 34.97222877080484, 'median': 35.0, 'width': 3.6999999999999957}, 'L_stats': {'mean': 35.12532916548262, 'median': 35.1, 'width': 3.700000000000003}, 'diff_mean': 0.1531003946777787, 'width_ref': 5, 'pad_band': 0.8, 'R_label': 'desna132.csv', 'L_label': 'leva132.csv'}
